# Supplementary material for: Purine and Purine Isostere Derivatives of Ferrocene: An Evaluation of ADME, Antitumor and Electrochemical Properties
Source: Molecules. 2020 Mar 29;25(7):1570. doi: 10.3390/molecules25071570 (PMC7180452; doi:10.3390/molecules25071570)

Supplementary Materials

Purine and Purine Isostere Derivatives of Ferrocene: An Evaluation of ADME, Antitumor and Electrochemical Properties

Valentina Rep [1], Martina Piškor [1], Helena Šimek [1], Petra Mišetić [2], Petra Grbčić [3], Jasna Padovan [2], Vesna Gabelica Marković [4], Dijana Jadreško [5], Krešimir Pavelić [6], Sandra Kraljević Pavelić [3] and Silvana Raić-Malić [1,*]

[1] Department of Organic Chemistry, Faculty of Chemical Engineering and Technology, University of Zagreb, Zagreb 10000, Croatia; vrep@fkit.hr (V.R.); mpiskor@fkit.hr (M.P.); hsimek@fkit.hr (H.Š.)

[2] Fidelta d.o.o., Zagreb 10000, Croatia; Petra.Misetic@fidelta.eu (P.M.); Jasna.Padovan@fidelta.eu (J.P.)

[3] Department of Biotechnology, Center for High-Throughput Technologies, University of Rijeka, Rijeka 51000, Croatia; petra.grbcic@biotech.uniri.hr (P.G.); sandrakp@biotech.uniri.hr (S.K.P.)

[4] International Relations Office, Faculty of Chemical Engineering and Technology, University of Zagreb, Zagreb 10000, Croatia; vesnagm@fkit.hr

[5] Division for Marine and Environmental Research, Ruđer Bošković Institute, Zagreb 10000, Croatia; djadresko@irb.hr

[6] Faculty of Medicine, Juraj Dobrila University of Pula, Pula 52100, Croatia; pavelic@unipu.hr

* Correspondence: sraic@fkit.hr; Tel.: +385-1-4597213

Figure 1. a) ^{1}H i b) ^{13}C-NMR of **11a**

a)

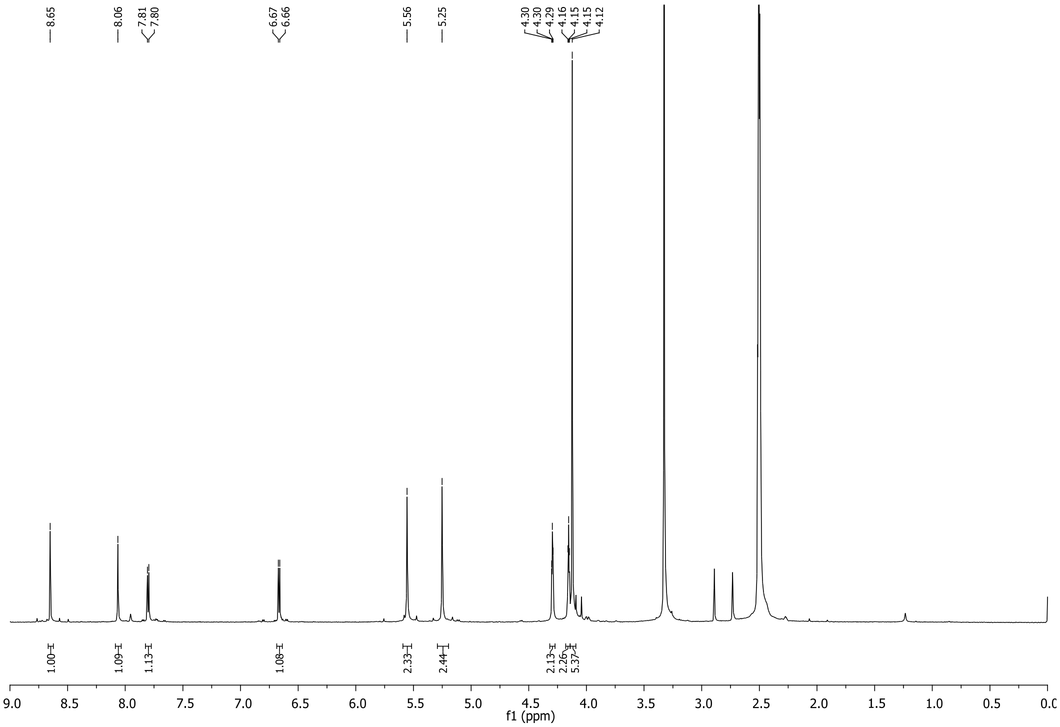

b)

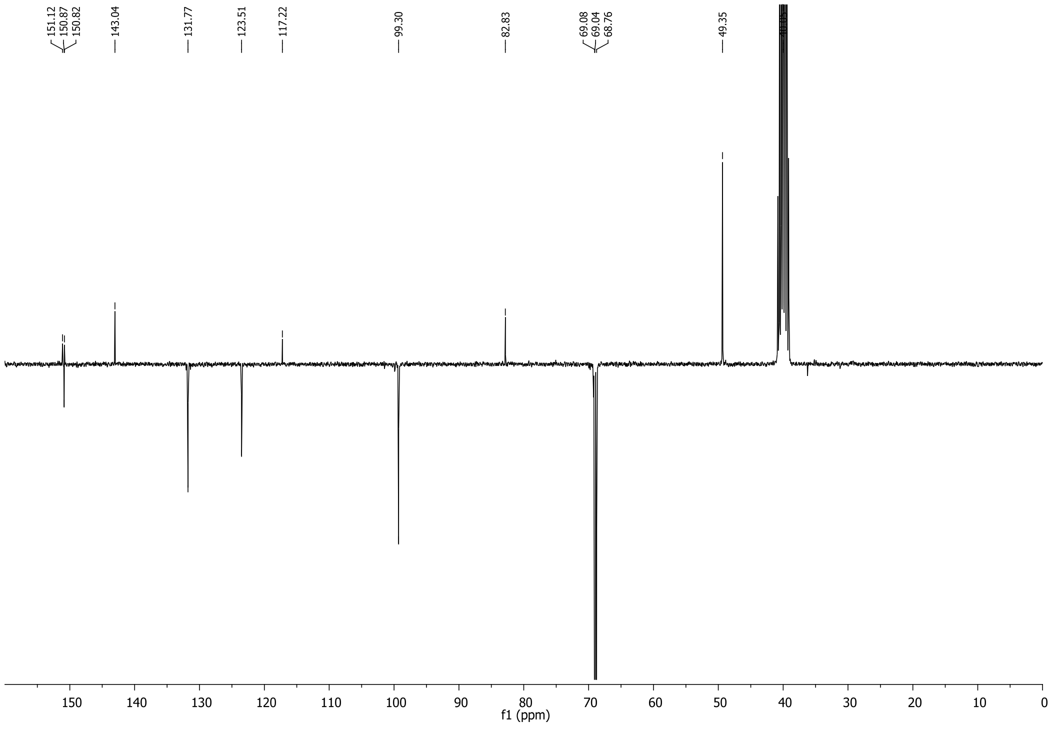

Figure S2. a) ^{1}H i b) ^{13}C-NMR of **11b**

a)

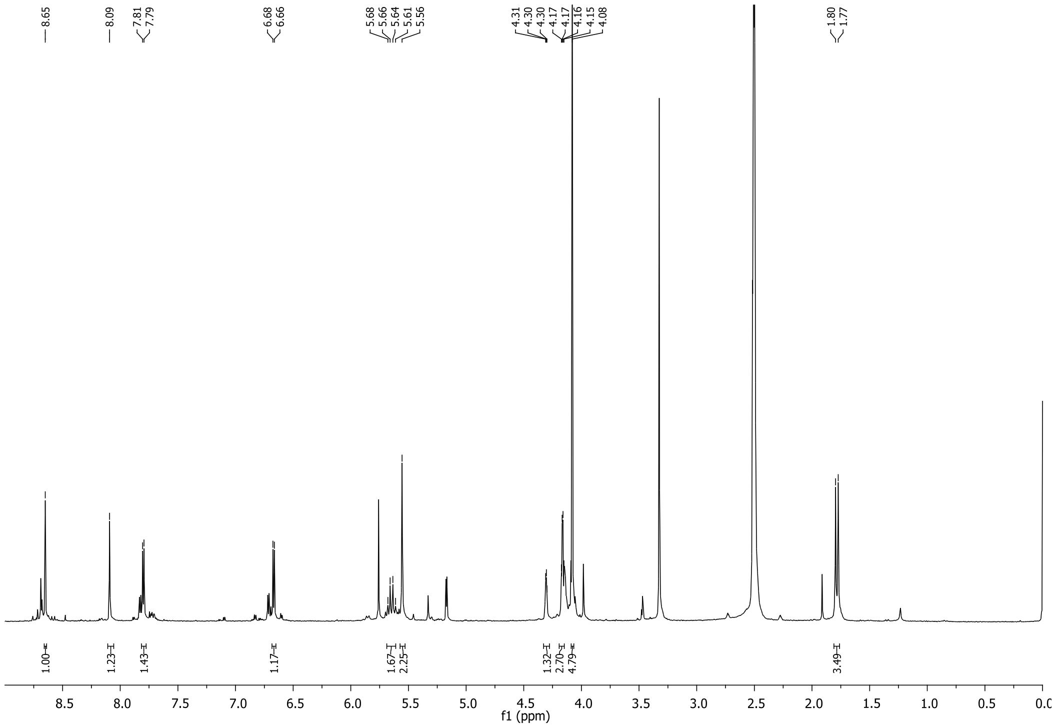

b)

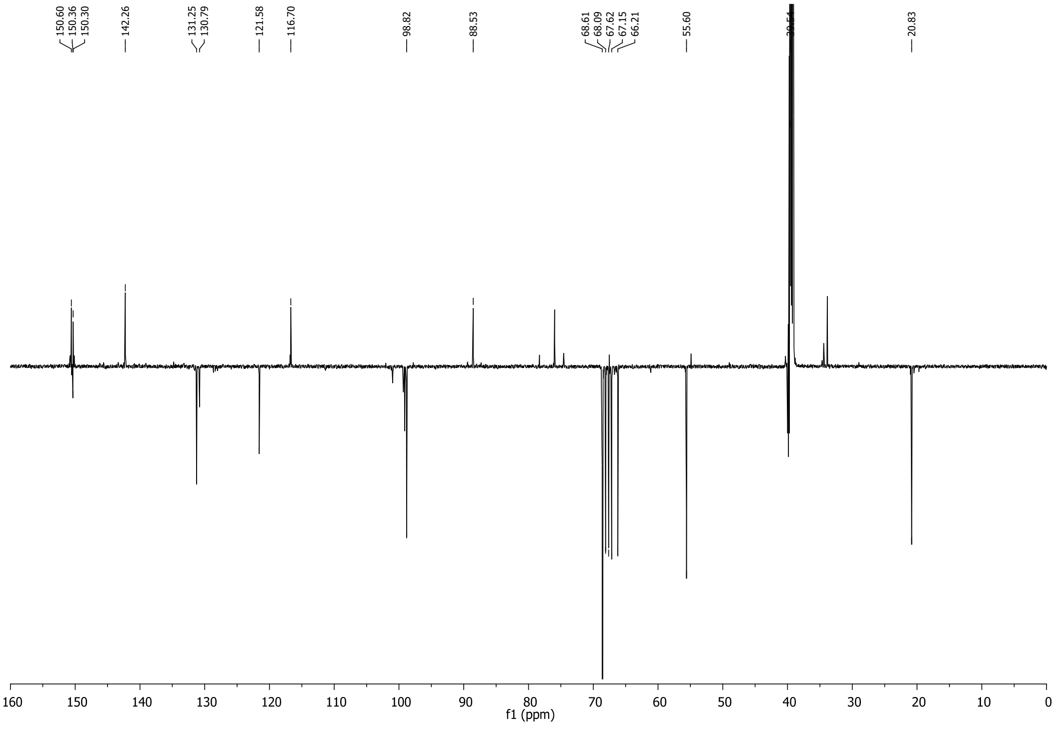

Figure S3. a) ^{1}H i b) ^{13}C-NMR of **11c**

a)

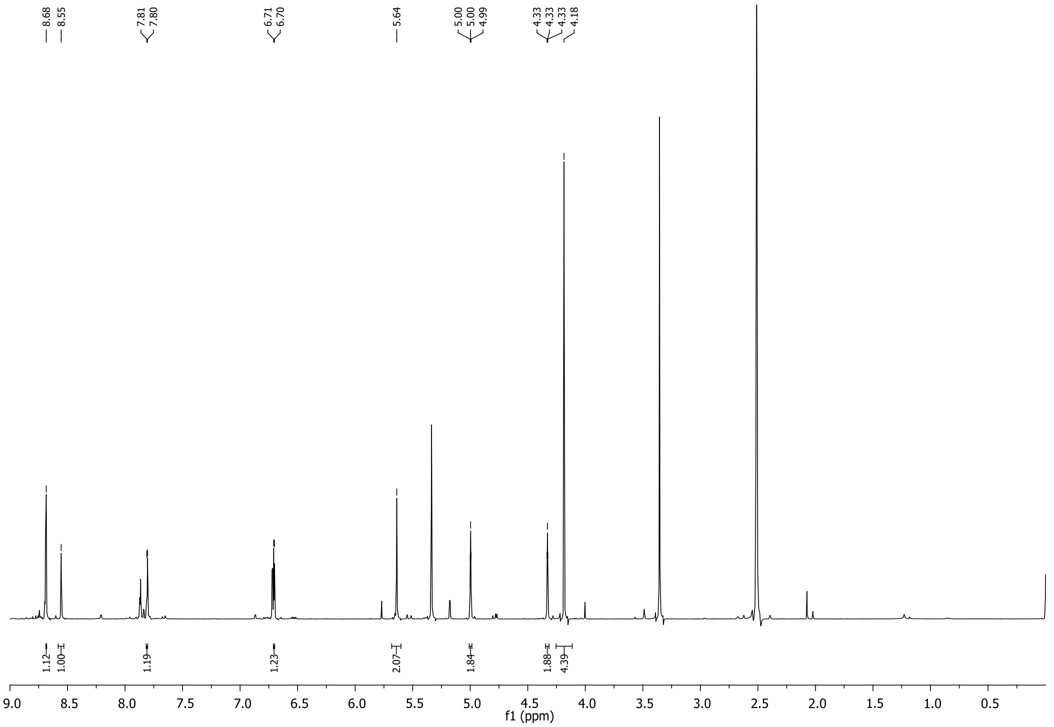

b)

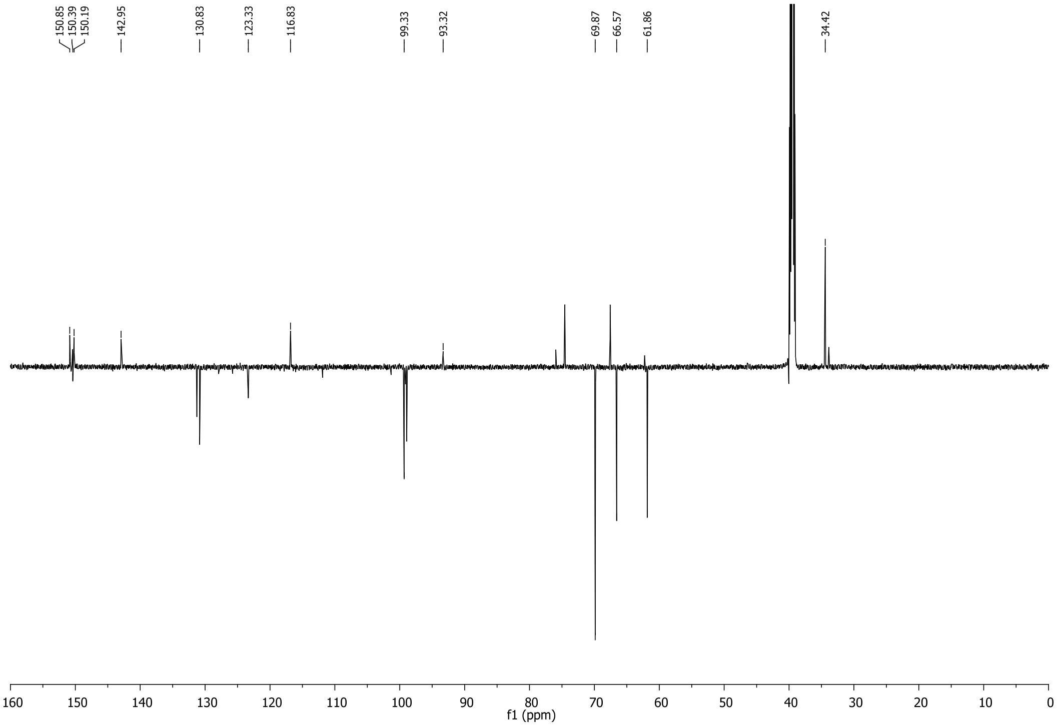

Figure S4. a) ^{1}H i b) ^{13}C-NMR of **12a**

a)

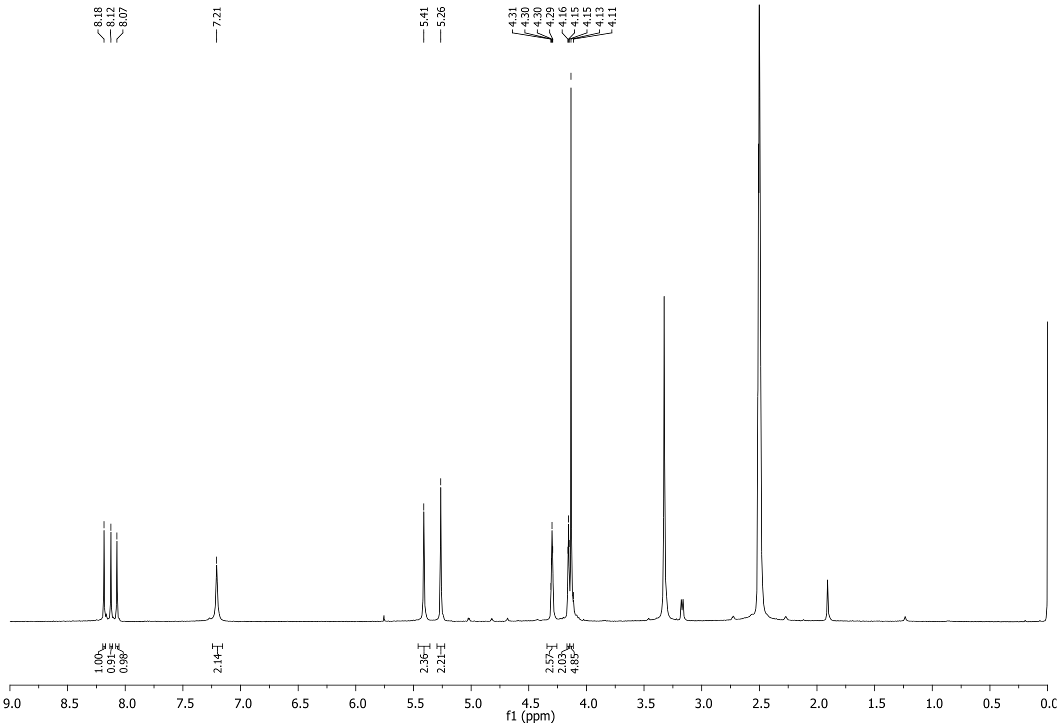

b)

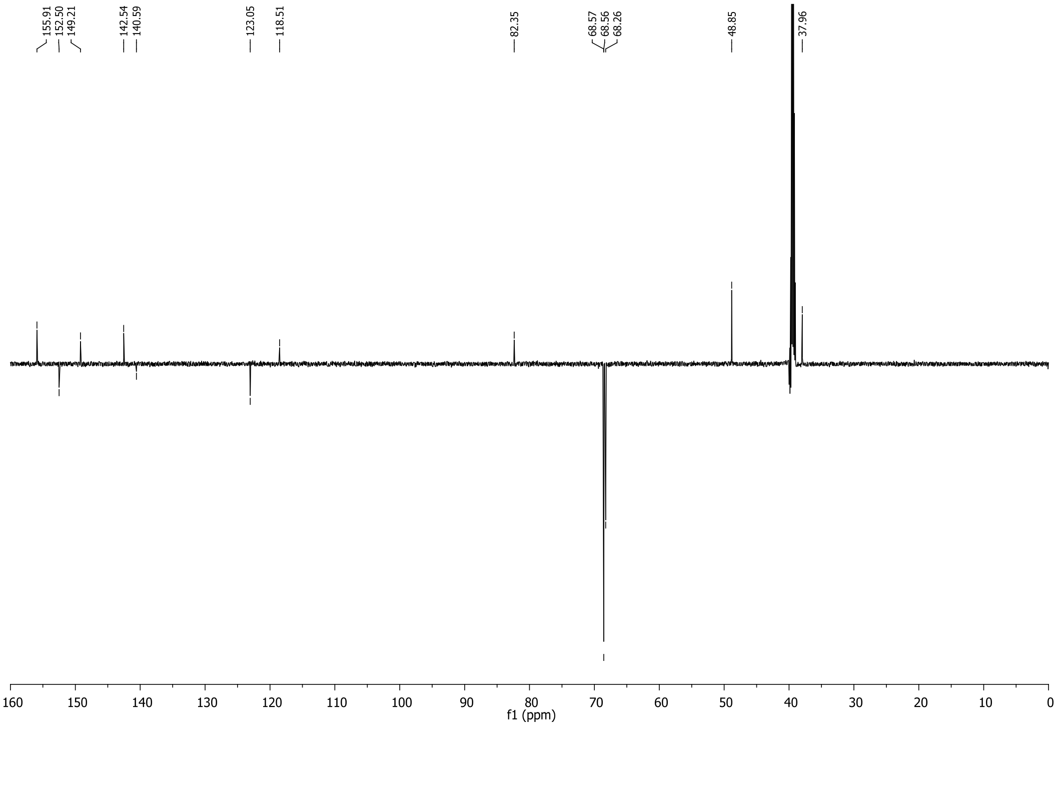

Figure S5. a) ^{1}H i b) ^{13}C-NMR of **12b**

a)

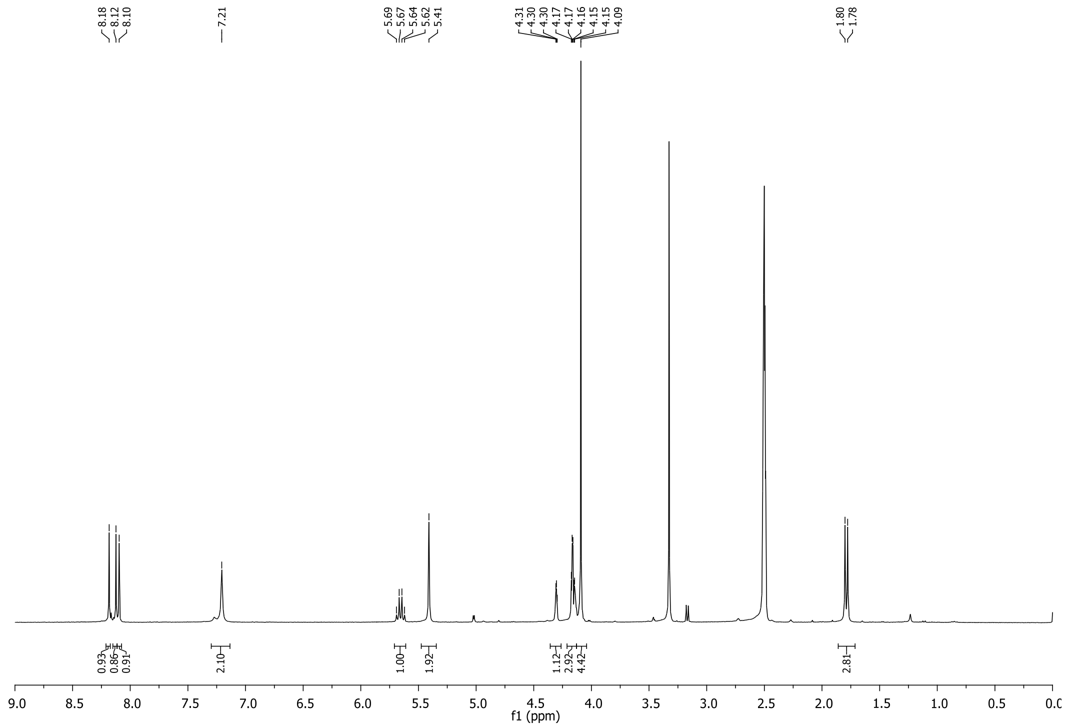

b)

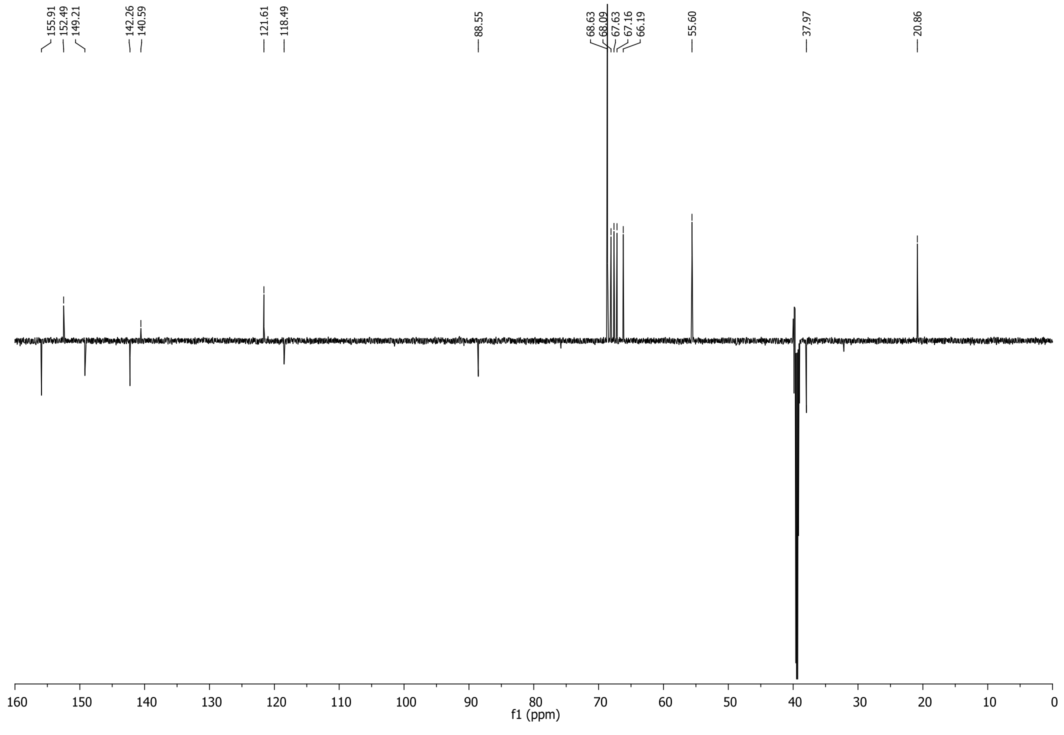

Figure S6. a) ^{1}H i b) ^{13}C-NMR of **12c**

a)

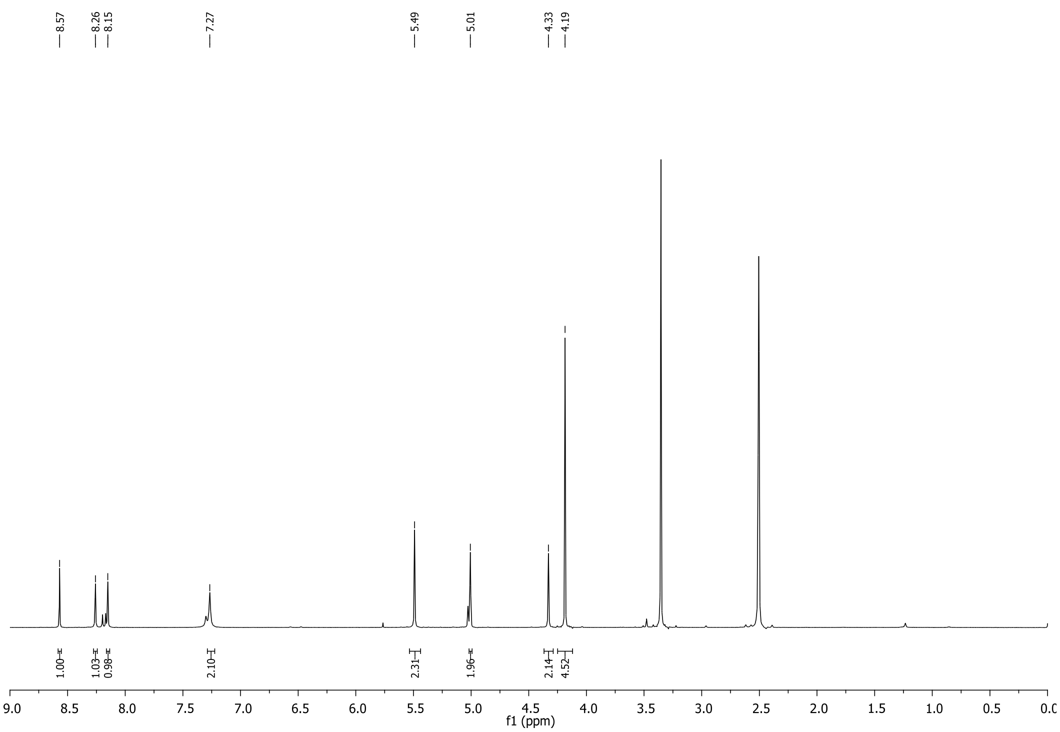

b)

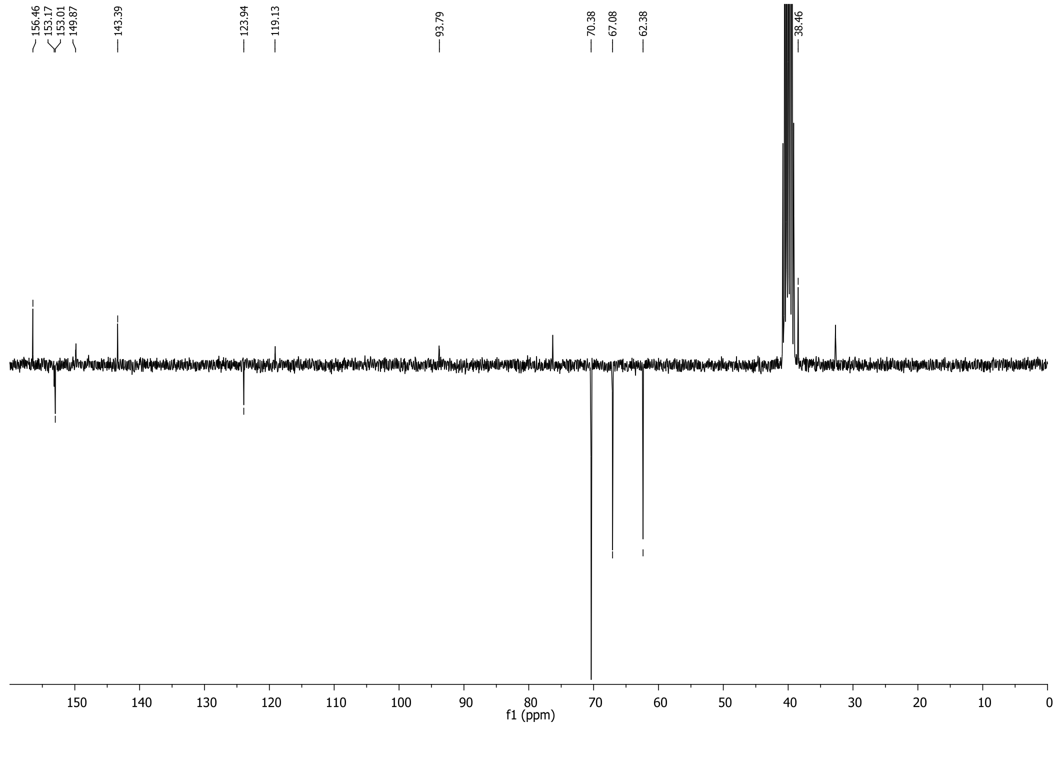

Figure S7. a) ^{1}H i b) ^{13}C-NMR of **13a**

a)

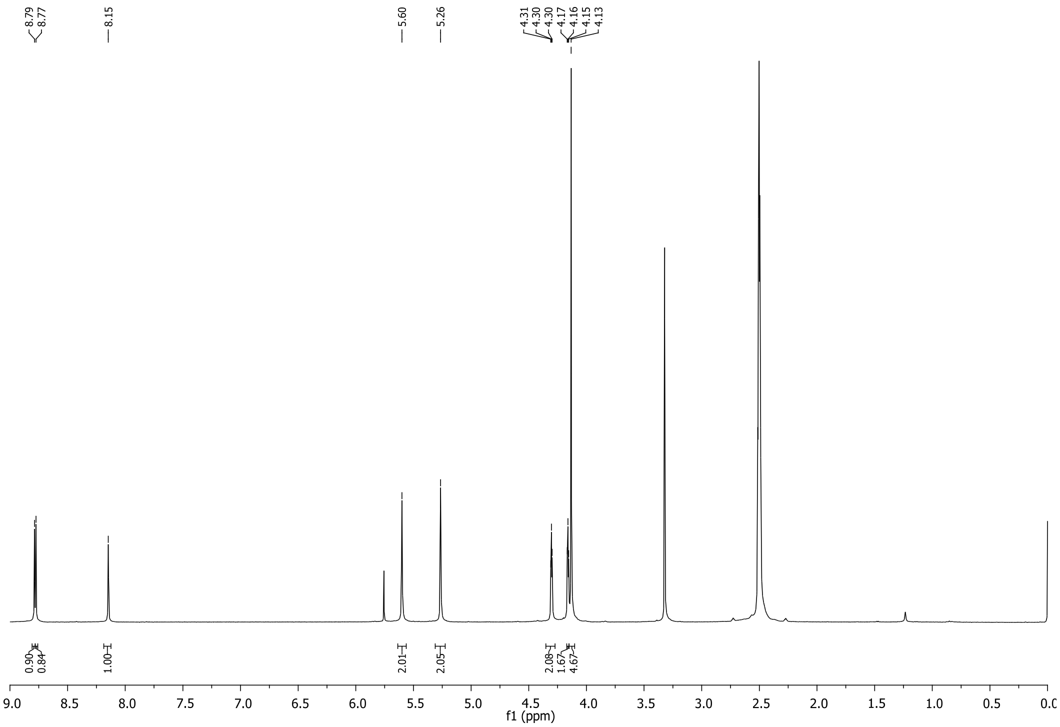

b)

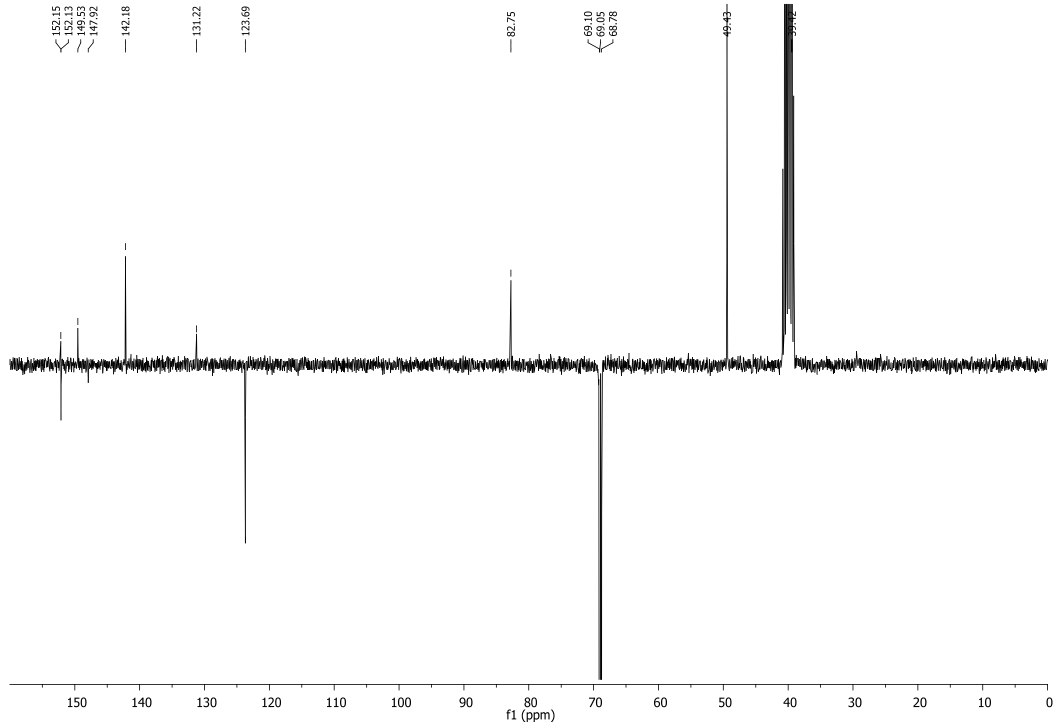

Figure S8. a) ^{1}H i b) ^{13}C-NMR of **13b**

a)

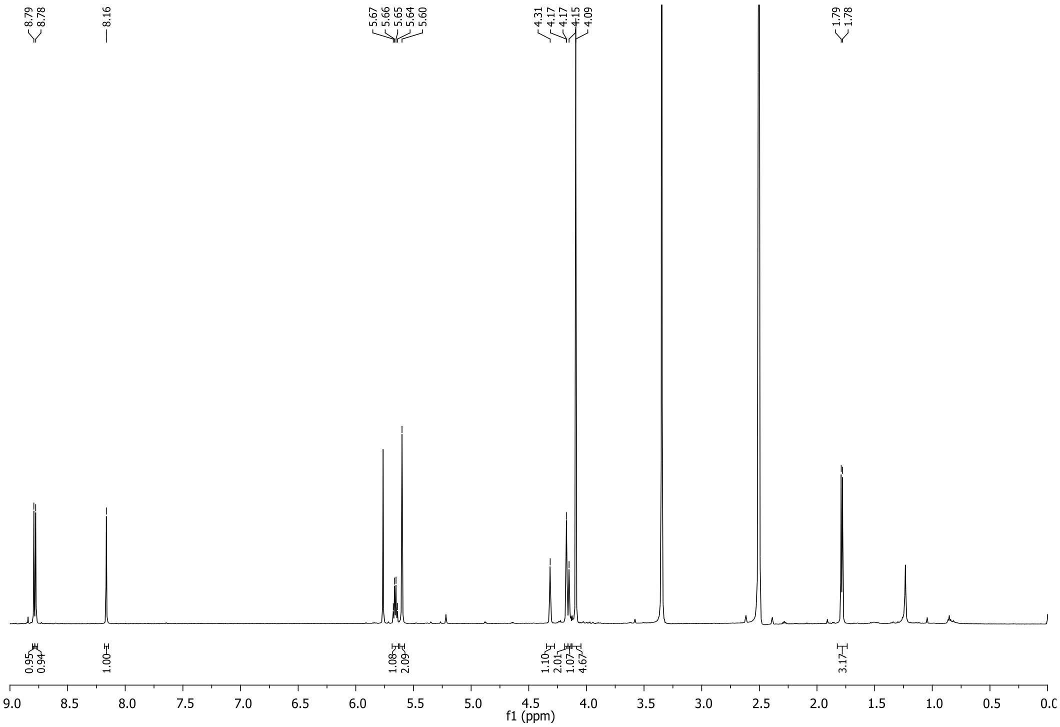

b)

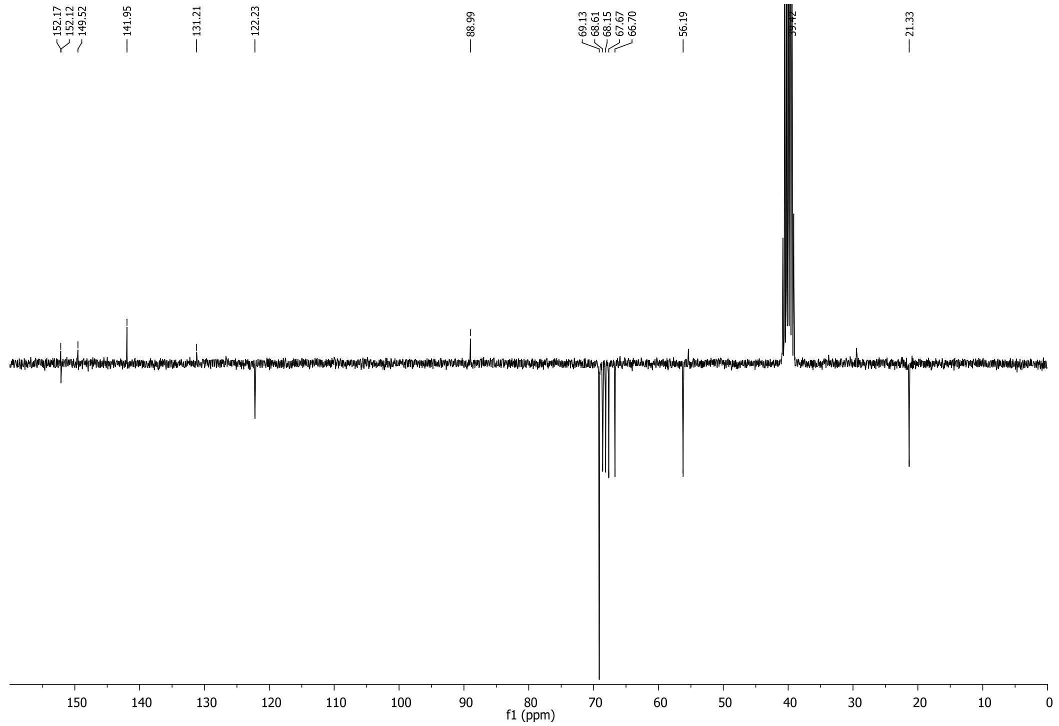

Figure S9. a) ^{1}H i b) ^{13}C-NMR of **13c**

a)

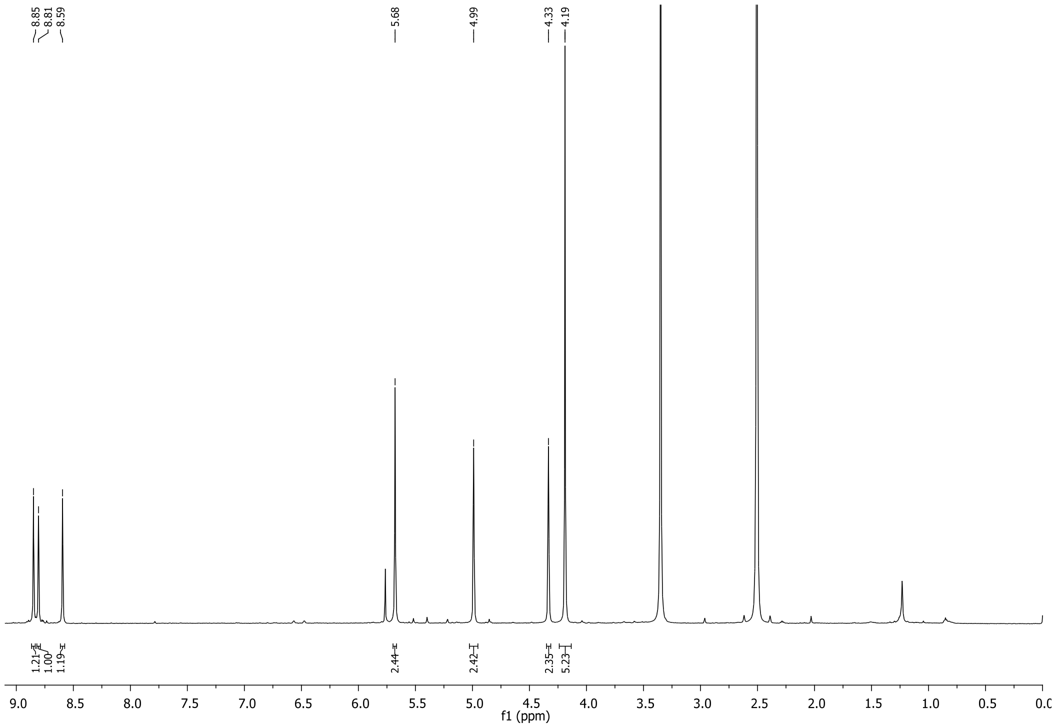

b)

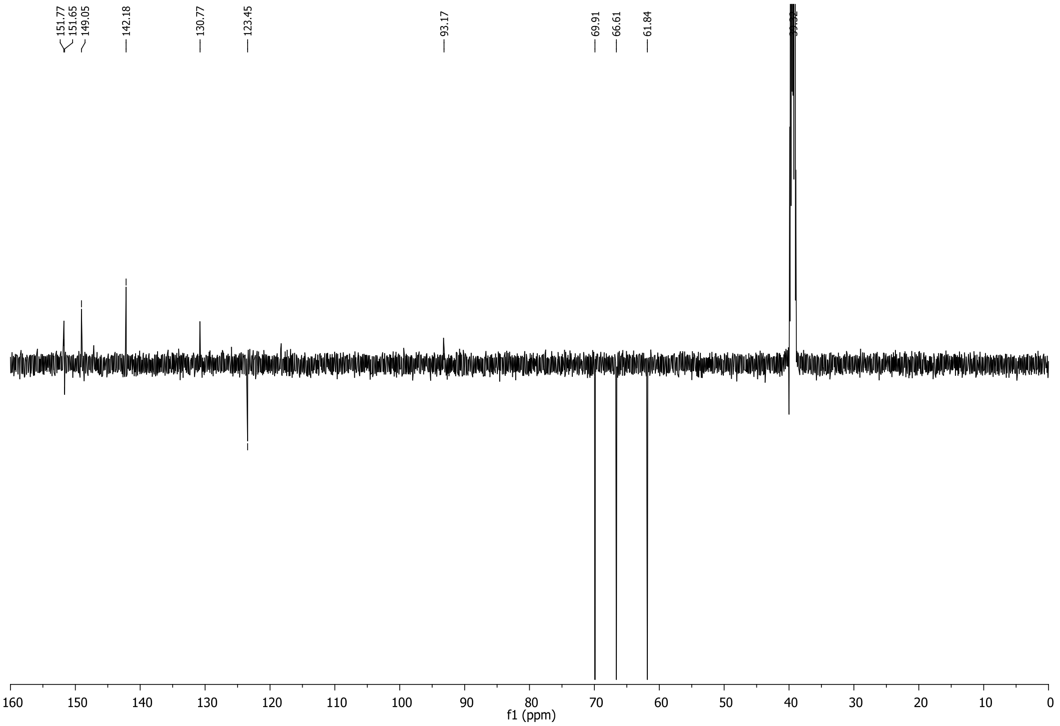

Figure S10. a) ^{1}H i b) ^{13}C-NMR of **14a**

a)

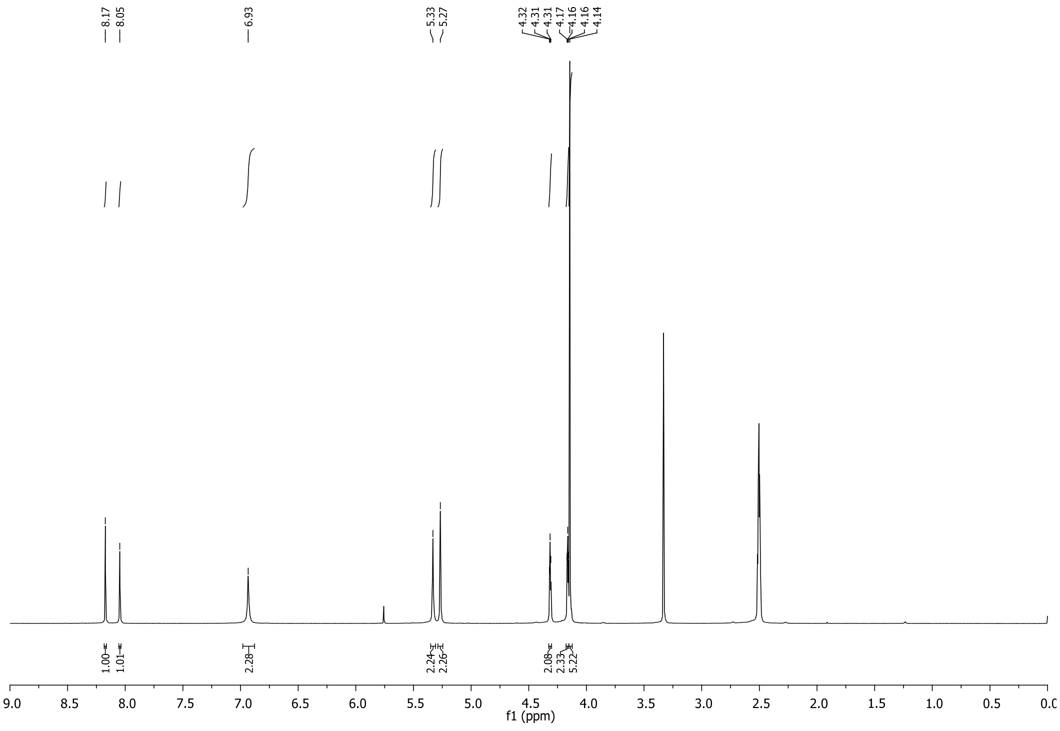

b)

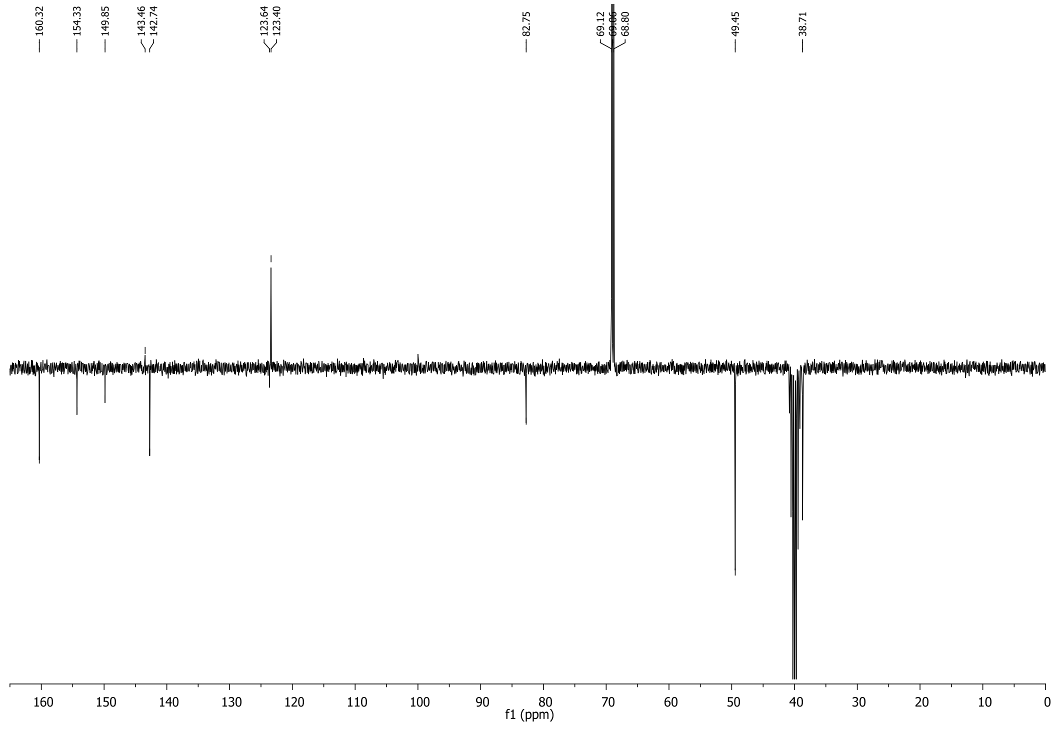

Figure S11. a) ^{1}H i b) ^{13}C-NMR of **14b**

a)

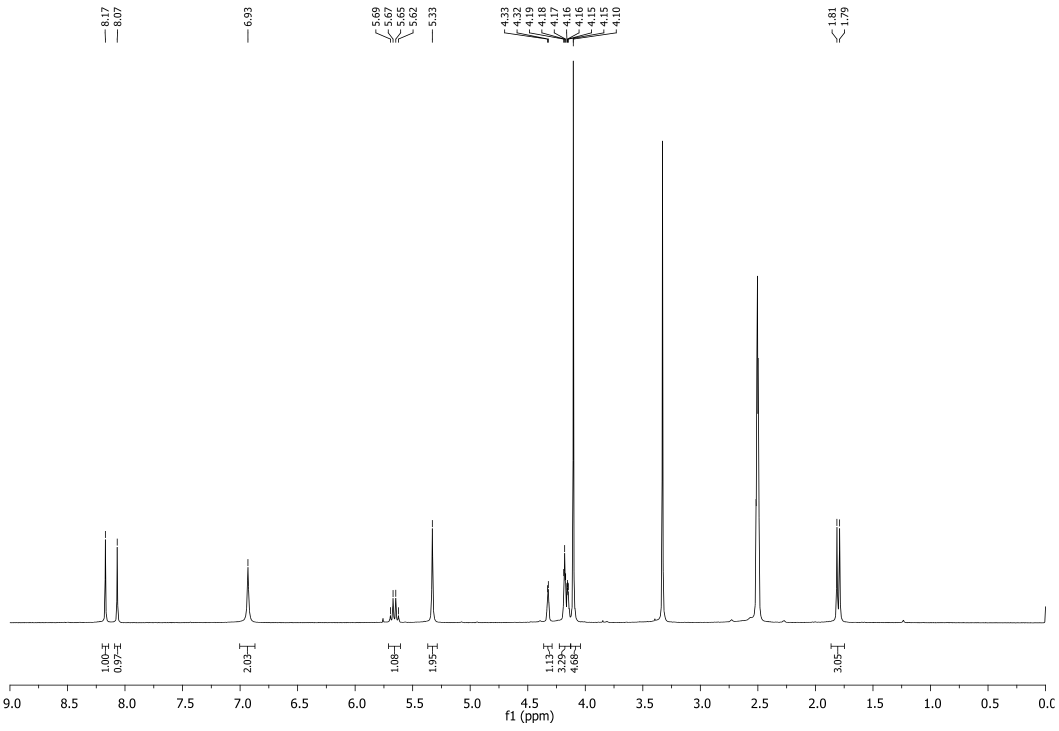

b)

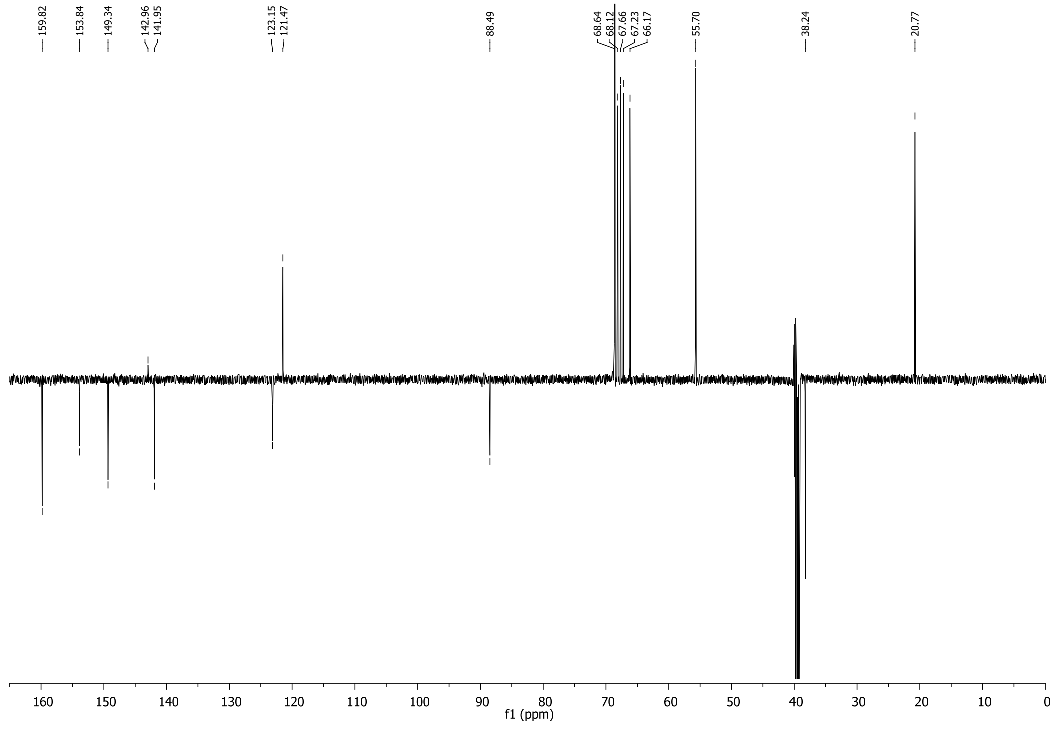

Figure S12. a) ^{1}H i b) ^{13}C-NMR of **14c**

a)

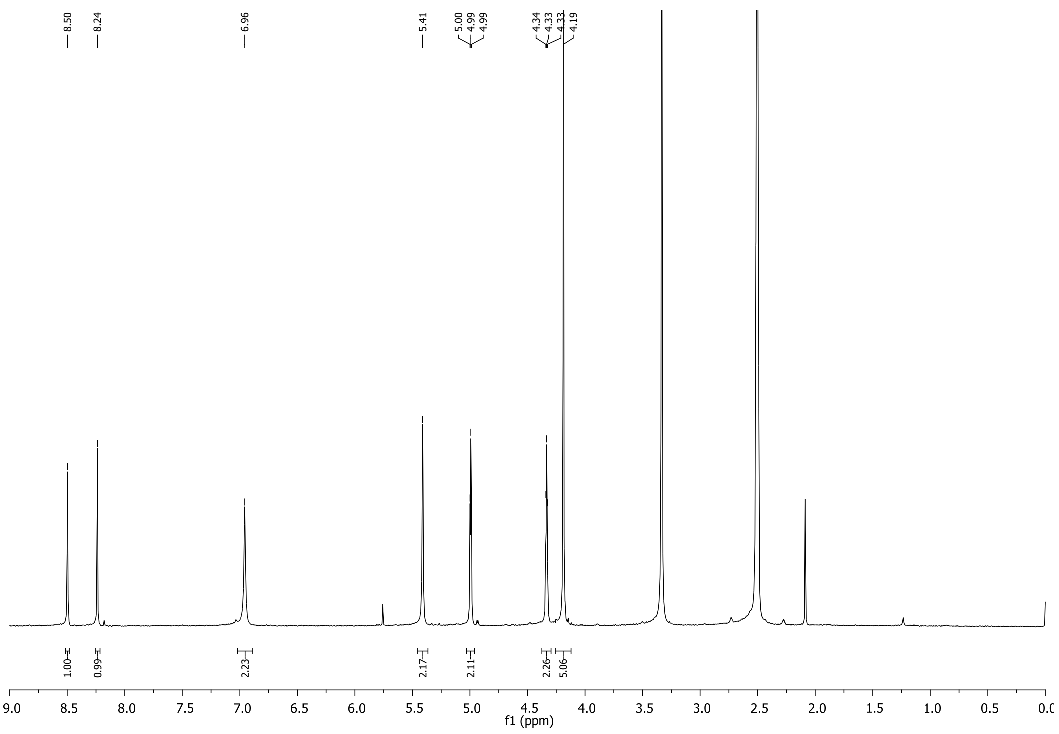

b)

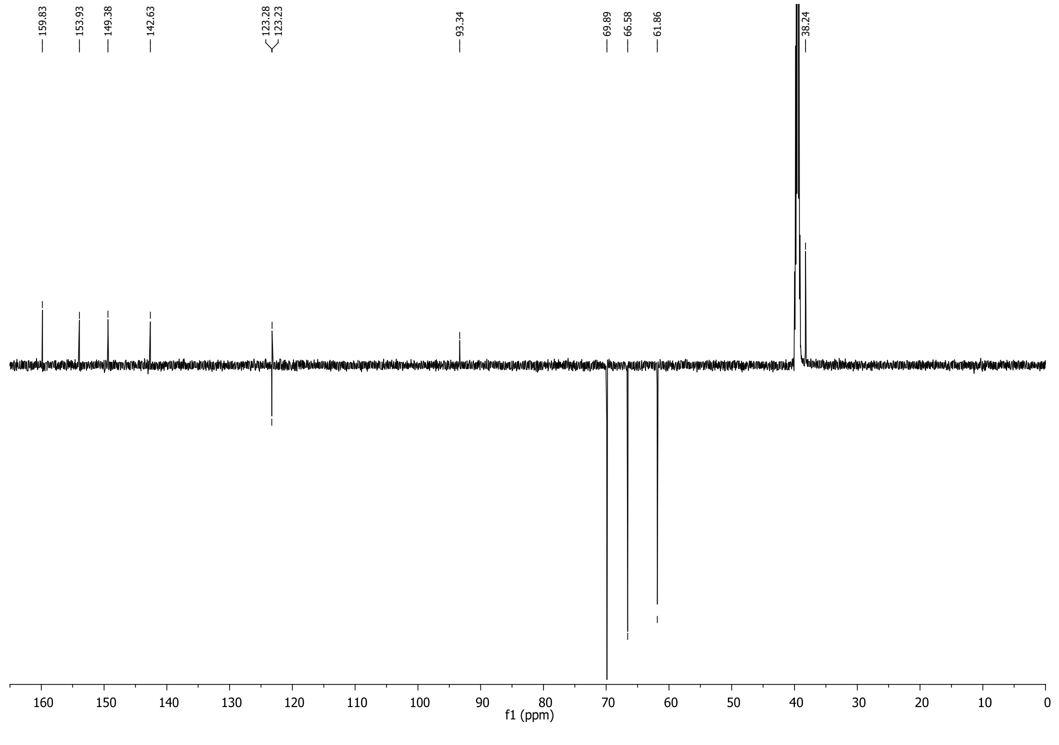

Figure S13. a) ^{1}H i b) ^{13}C-NMR of **15a**

a)

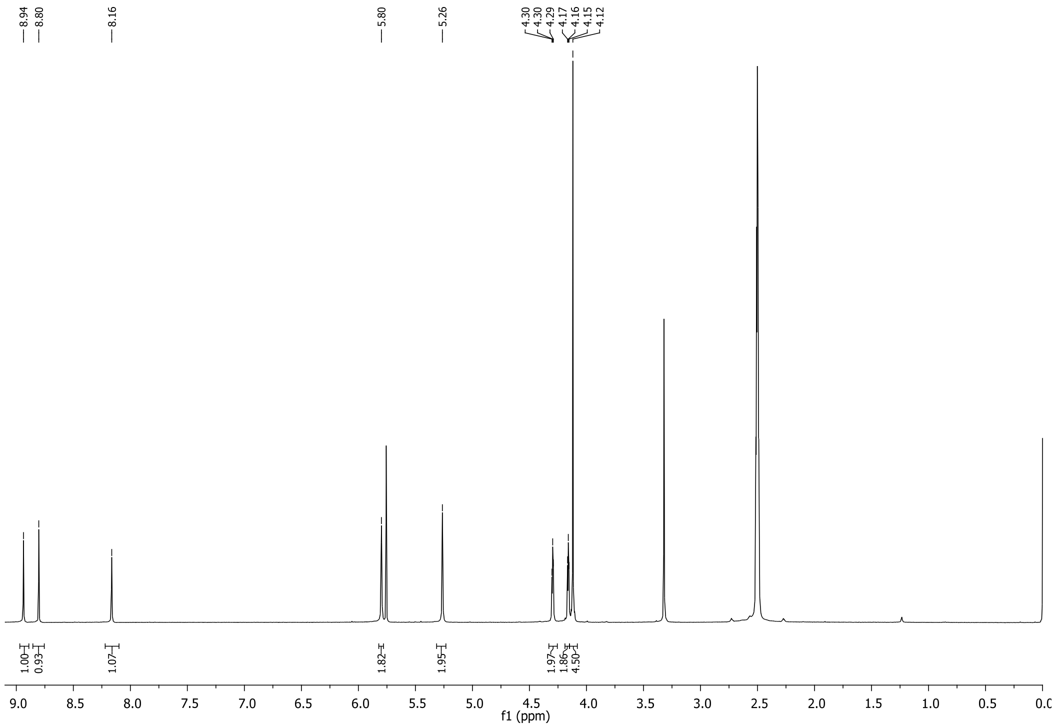

b)

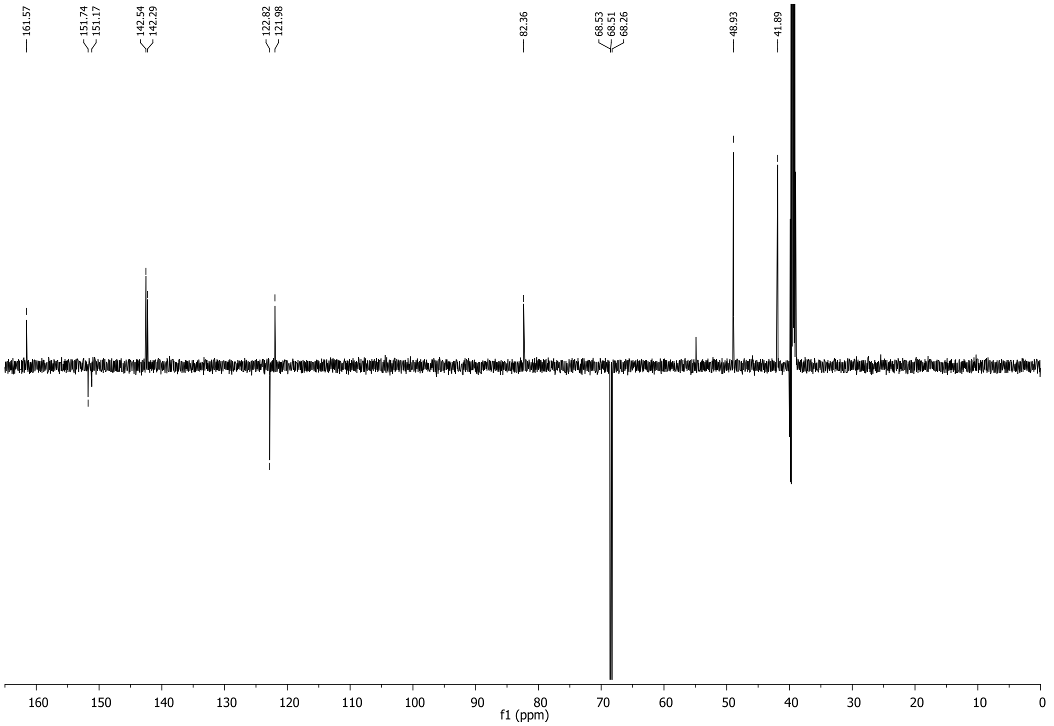

Figure S14. a) ^{1}H i b) ^{13}C-NMR of **15b**

a)

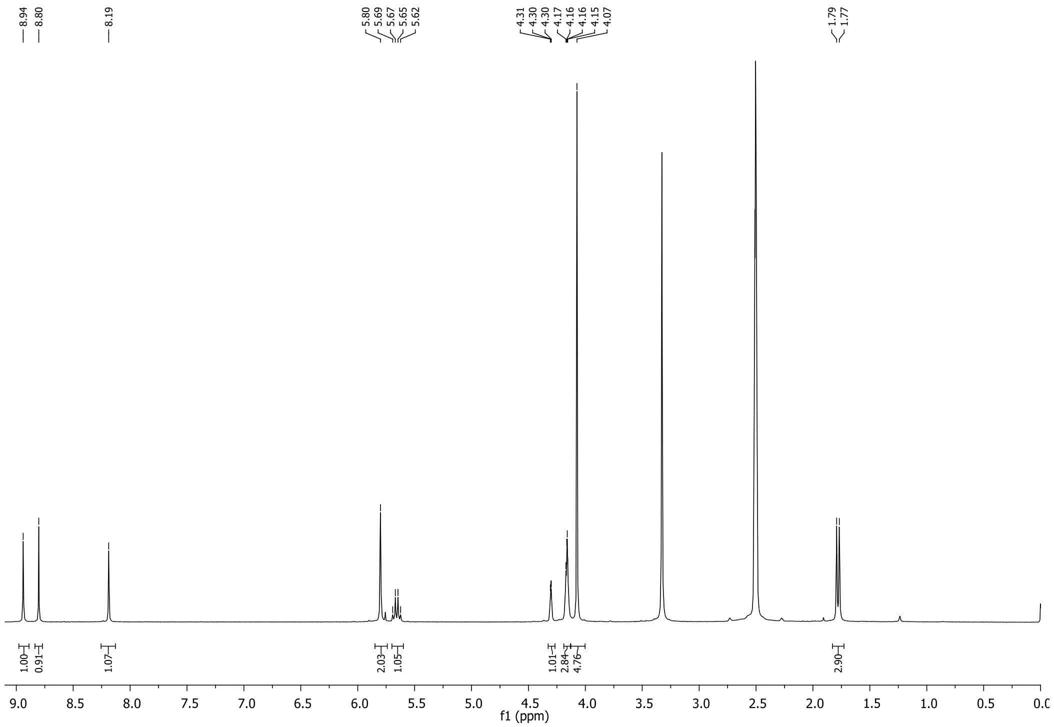

b)

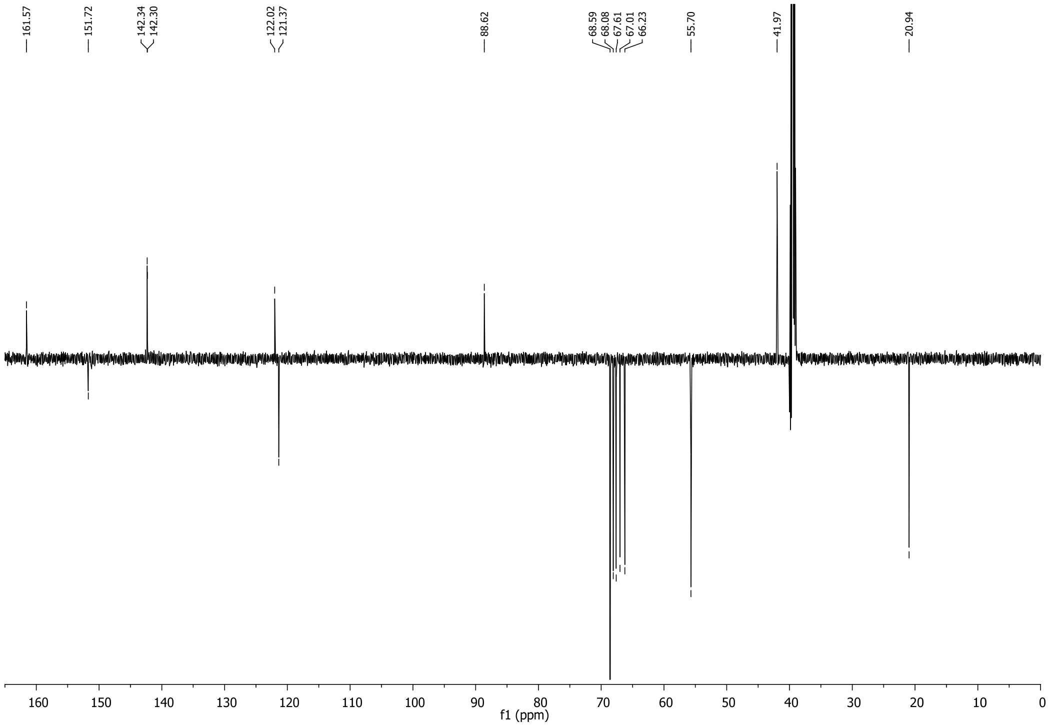

Figure S15. a) ^{1}H i b) ^{13}C-NMR of **15c**

a)

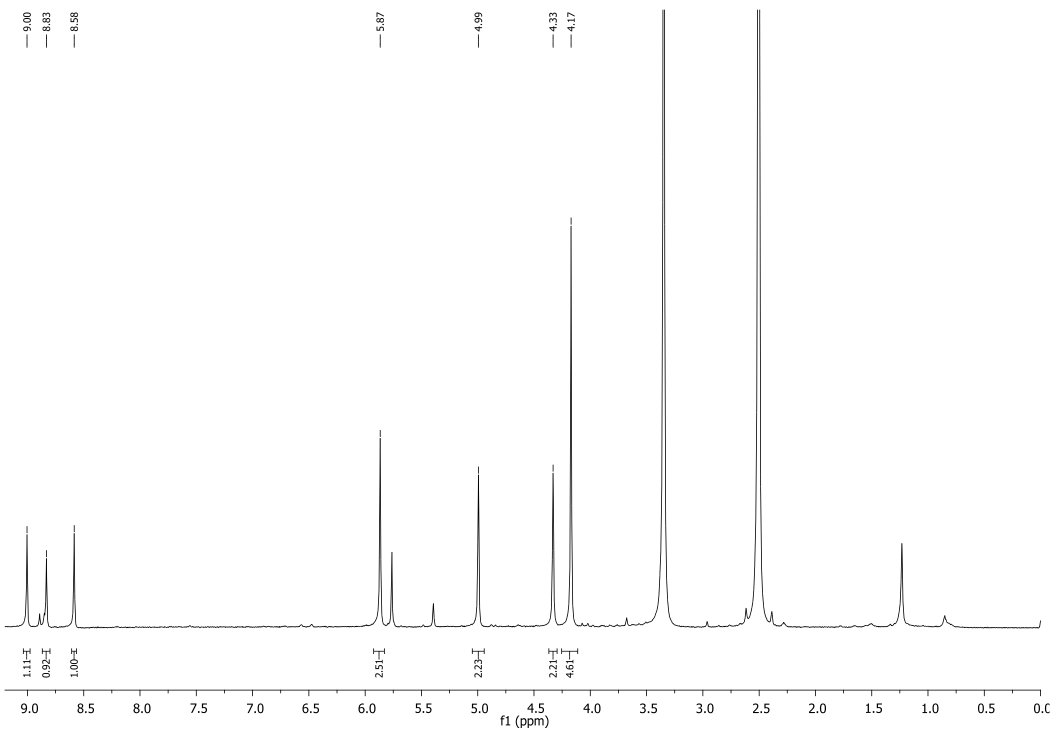

b)

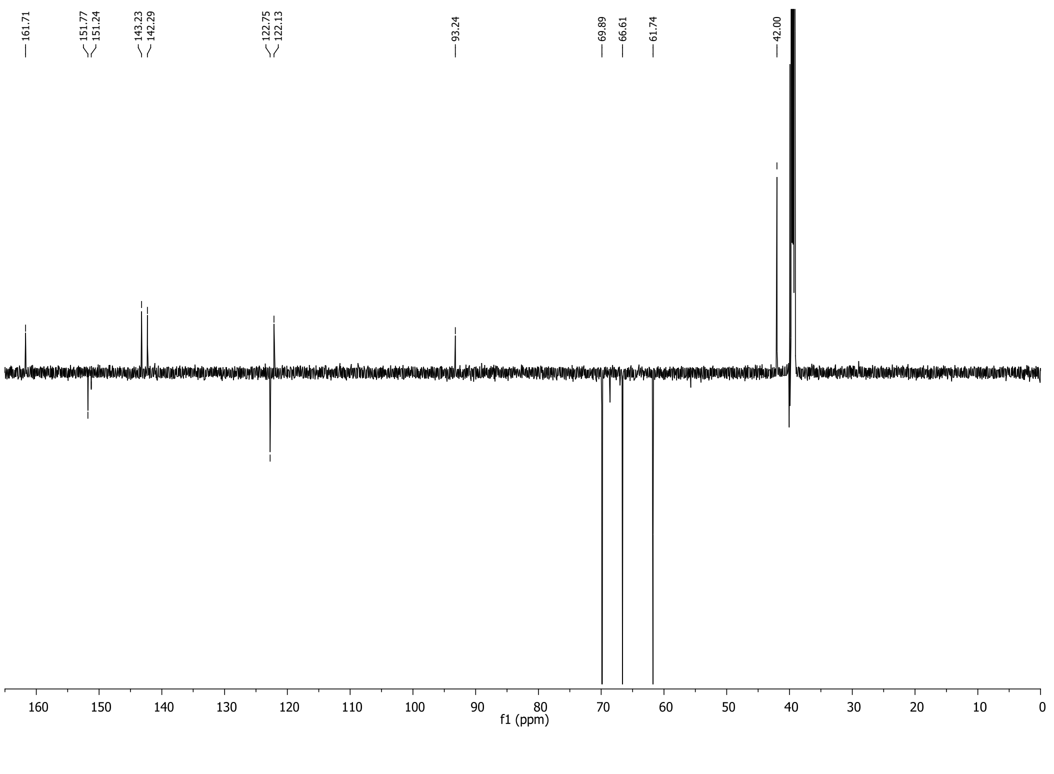

Figure S16. a) ^{1}H i b) ^{13}C-NMR of **16a**

a)

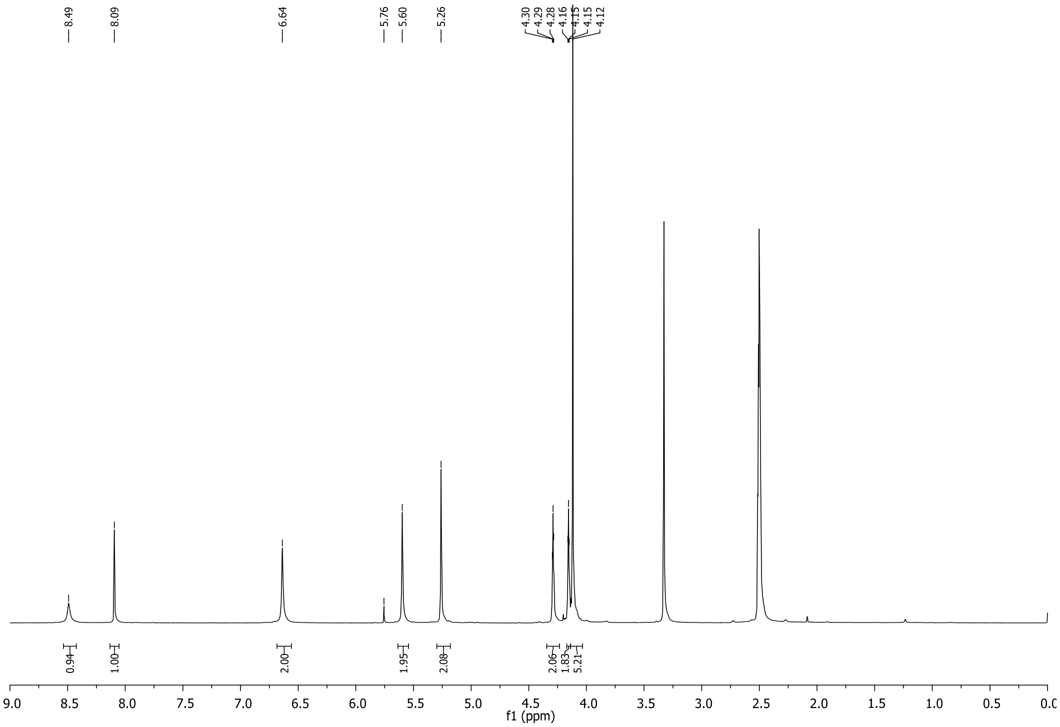

b)

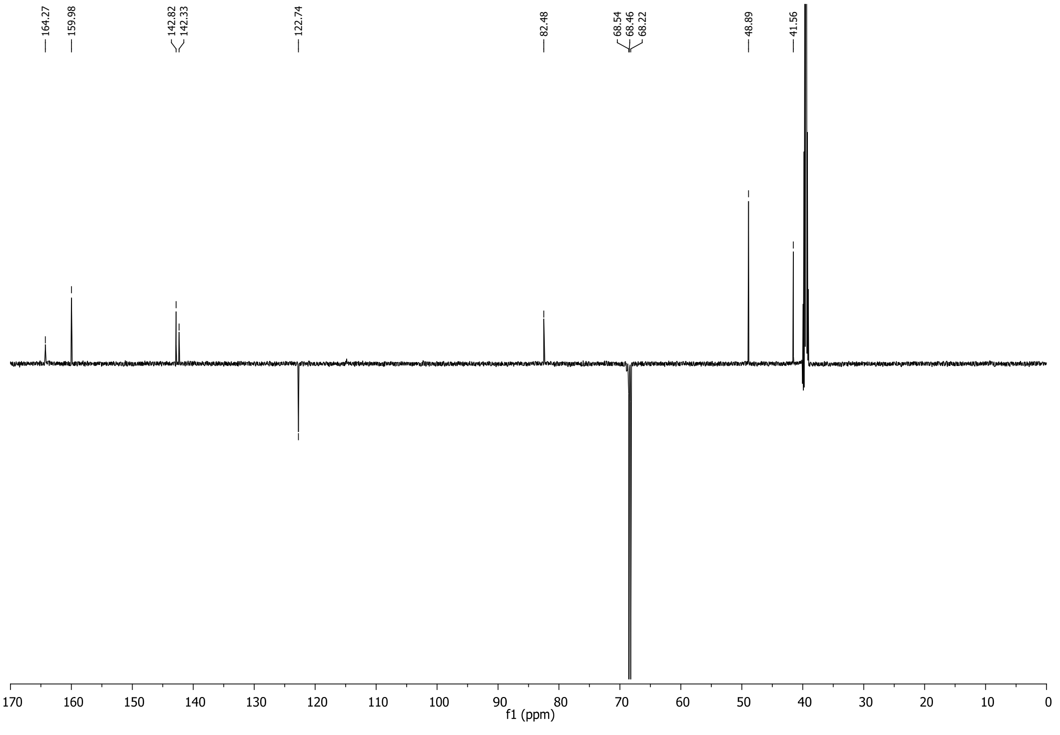

Figure S17. a) ^{1}H i b) ^{13}C-NMR of **23a**

a)

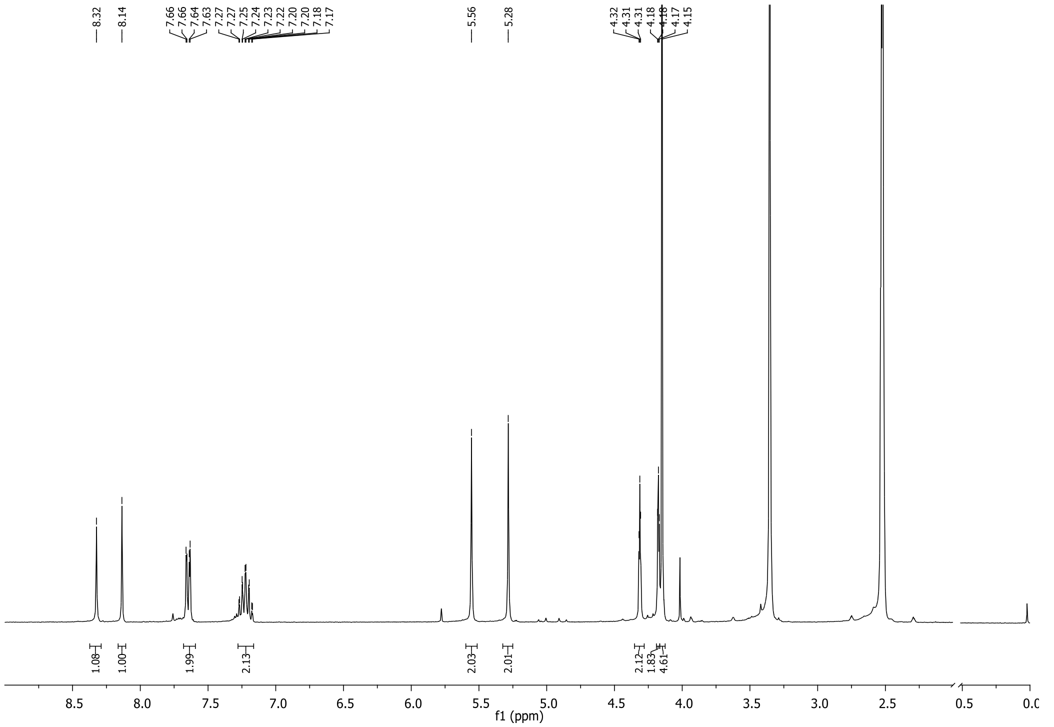

b)

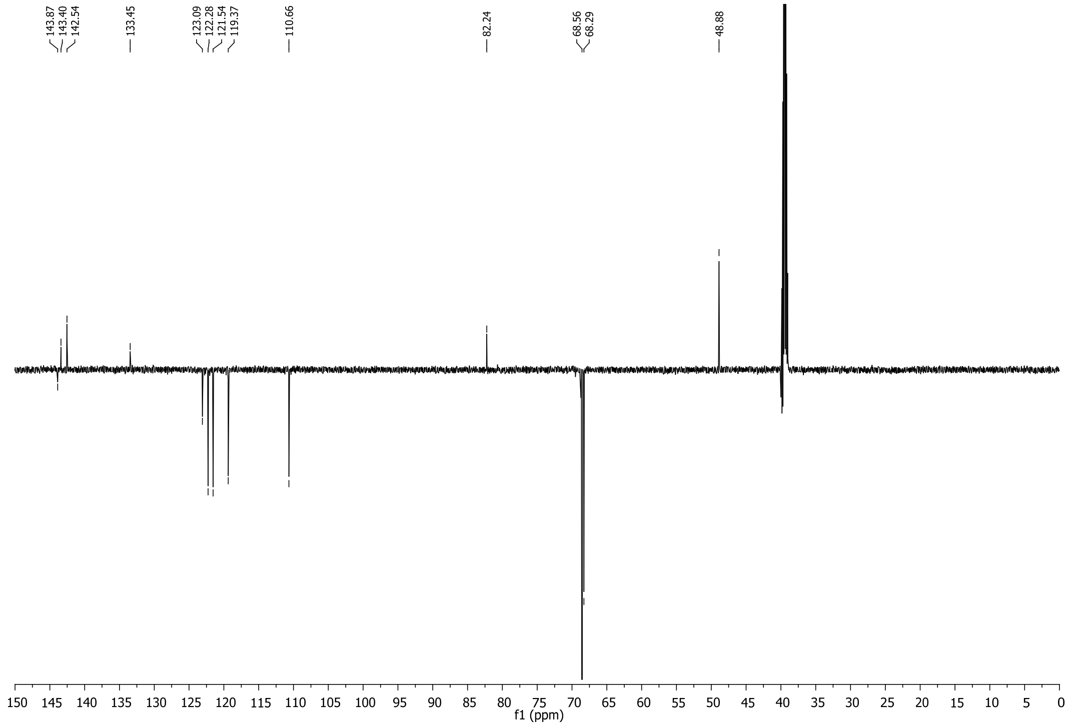

Figure S18. a) ^{1}H i b) ^{13}C-NMR of **23b**

a)

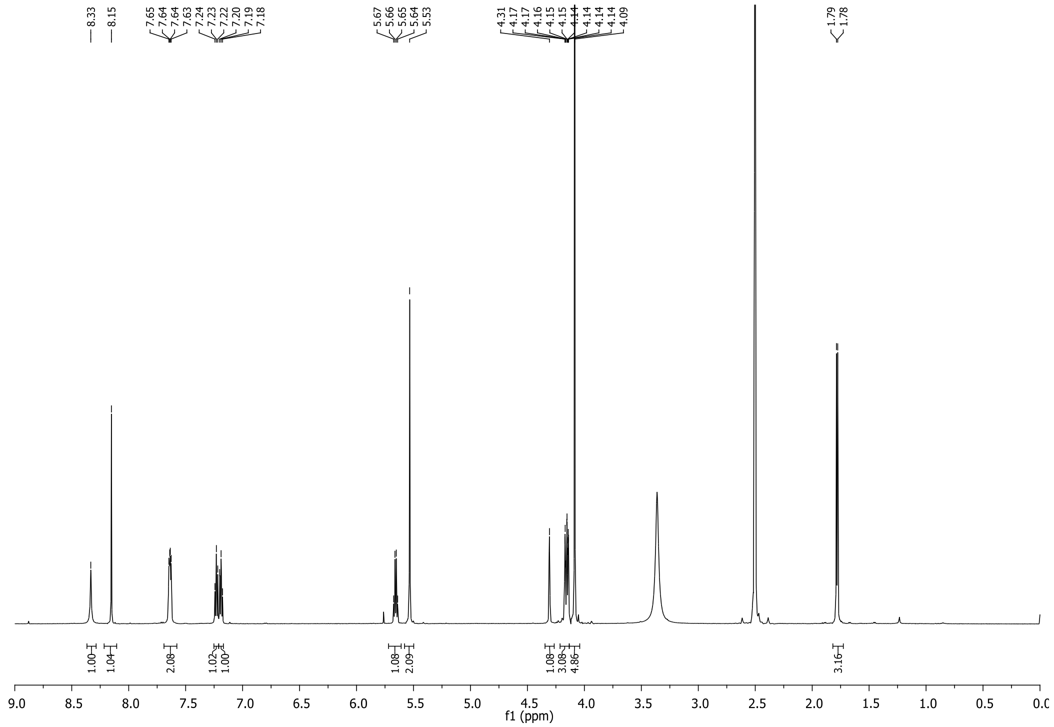

b)

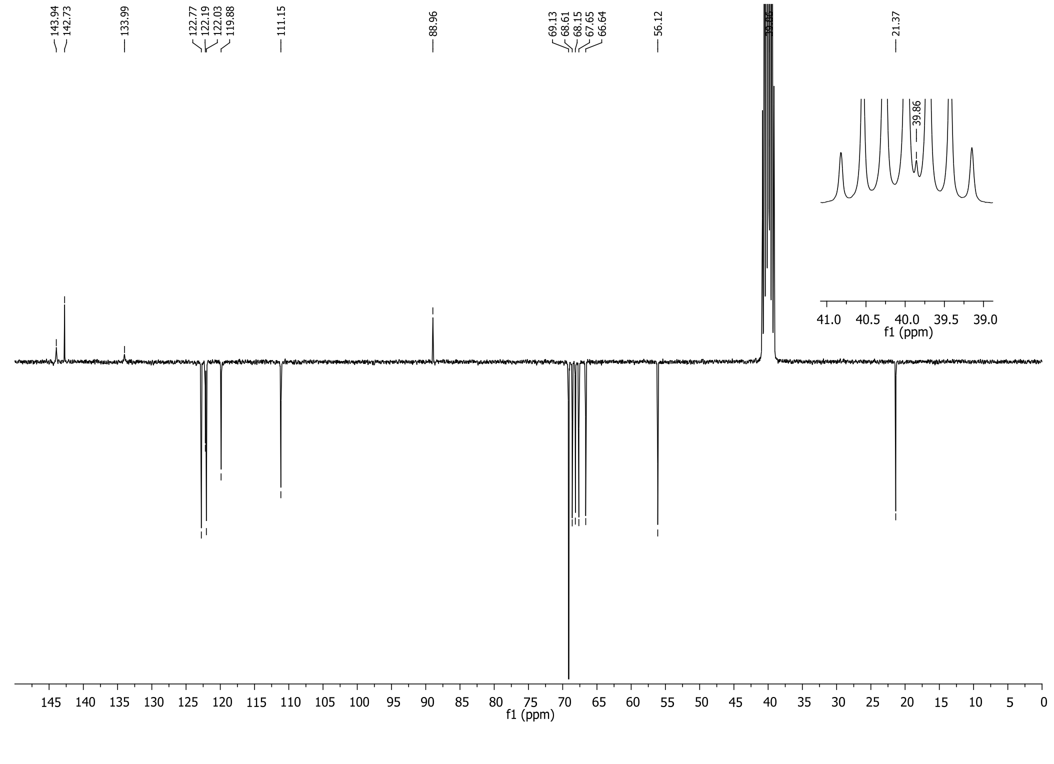

Figure S19. a) ^{1}H i b) ^{13}C-NMR of **23c**

a)

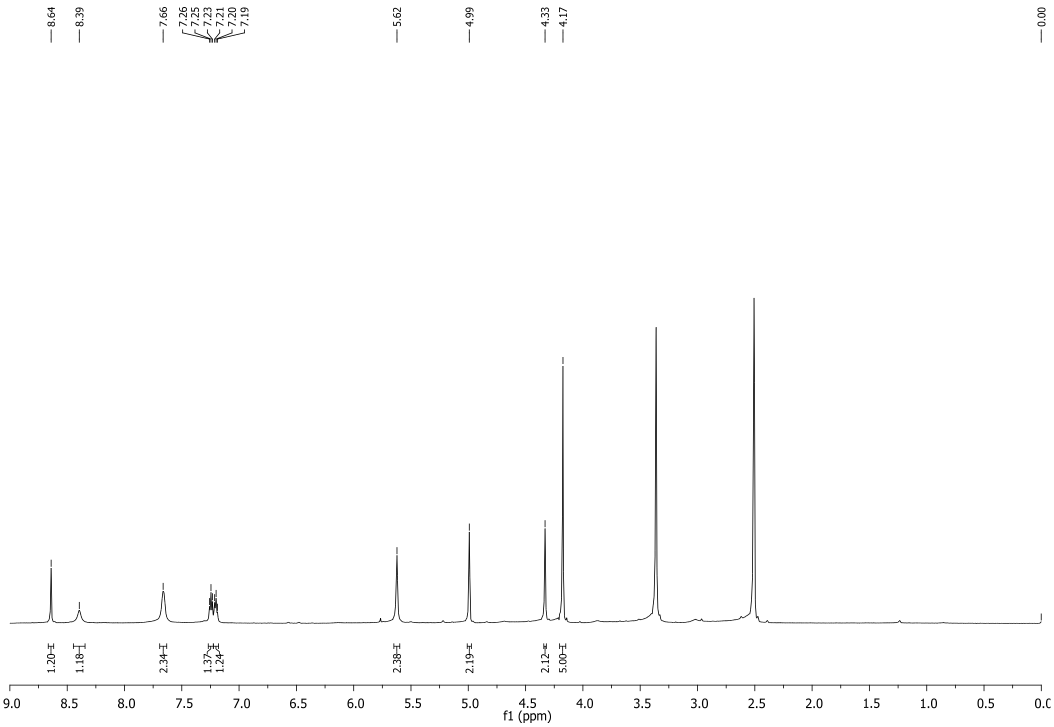

b)

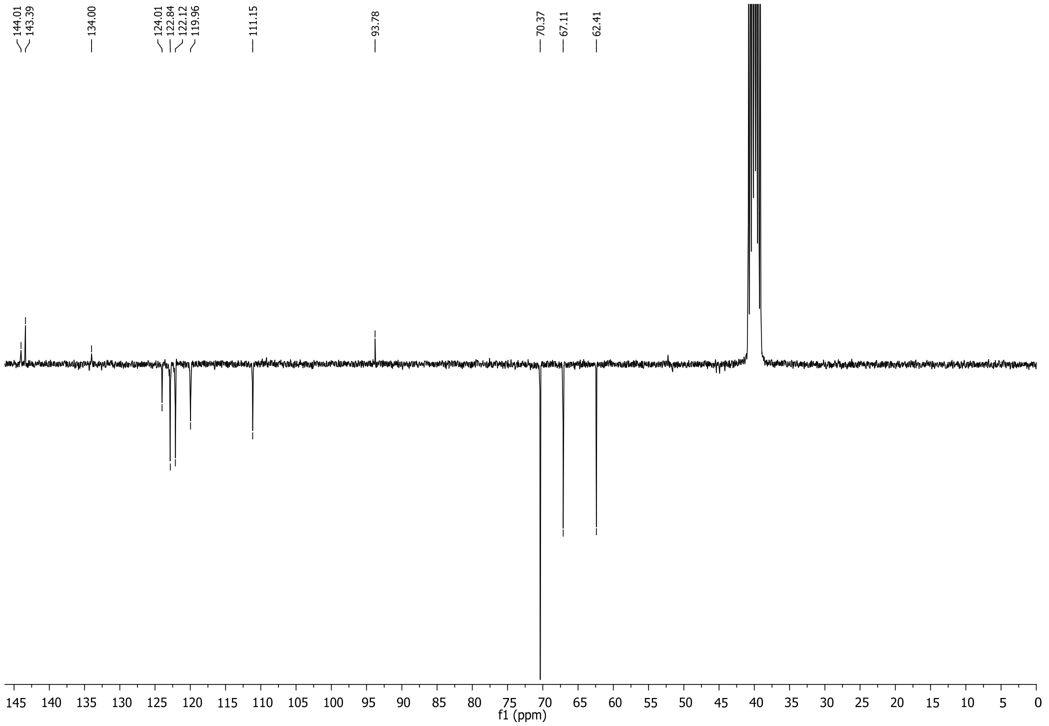

Figure S20. a) ^{1}H i b) ^{13}C-NMR of **24a**

a)

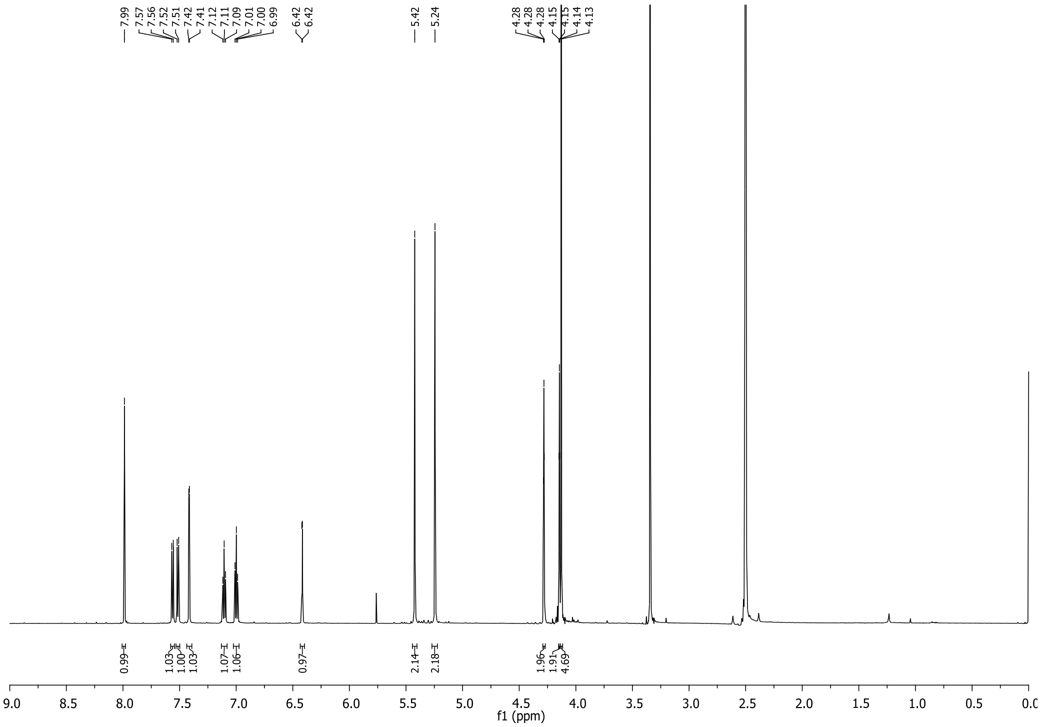

b)

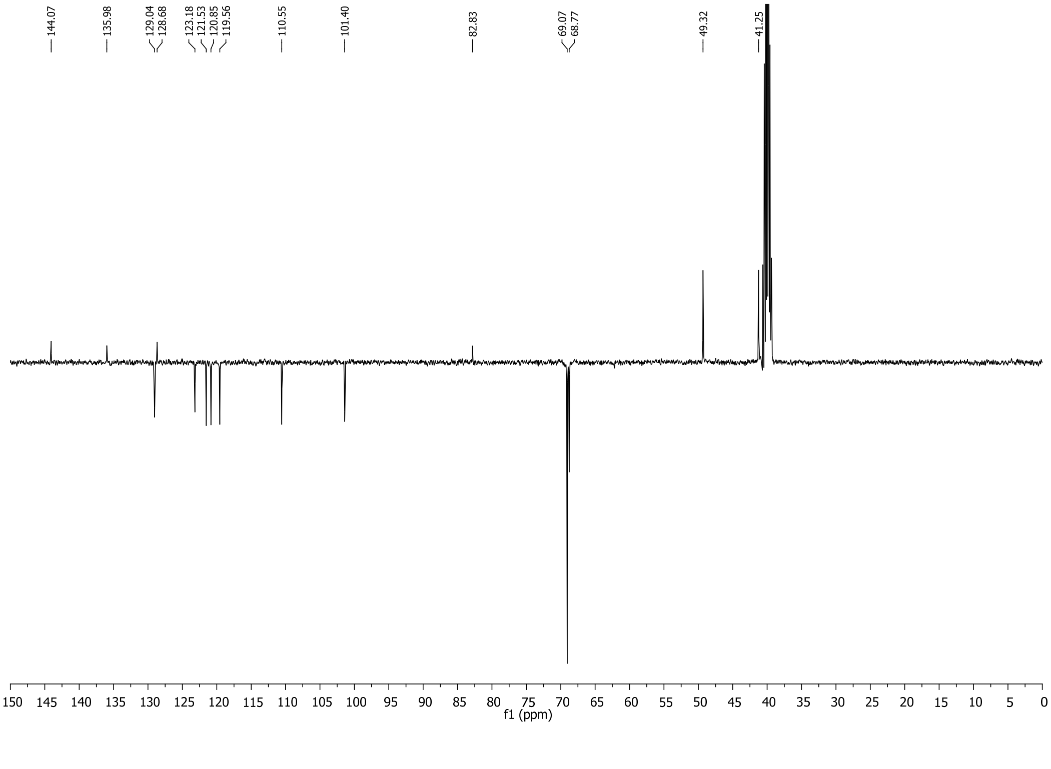

Figure S21. a) ^{1}H i b) ^{13}C-NMR of **24b**

a)

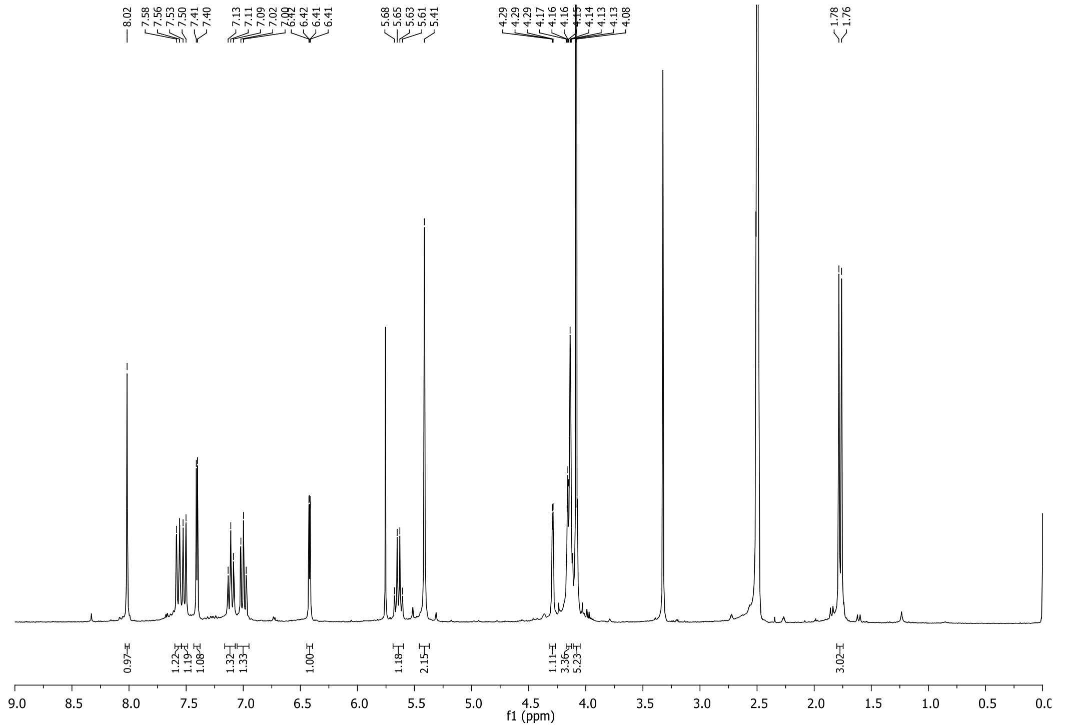

b)

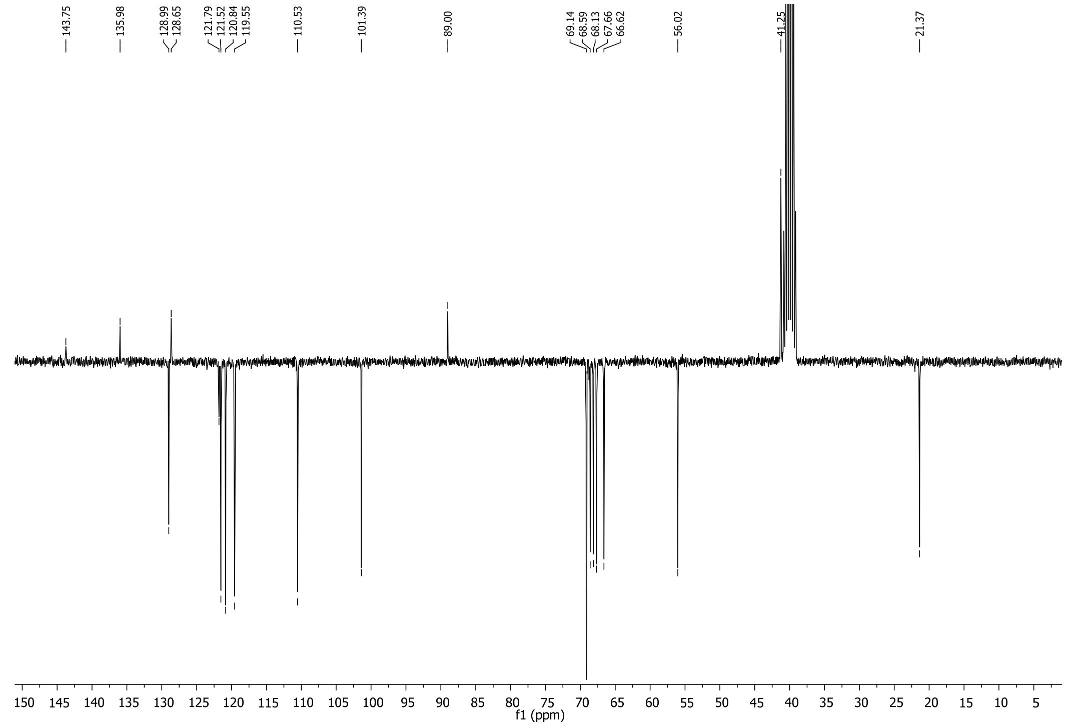

Figure S22. a) ^{1}H i b) ^{13}C-NMR of **24c**

a)

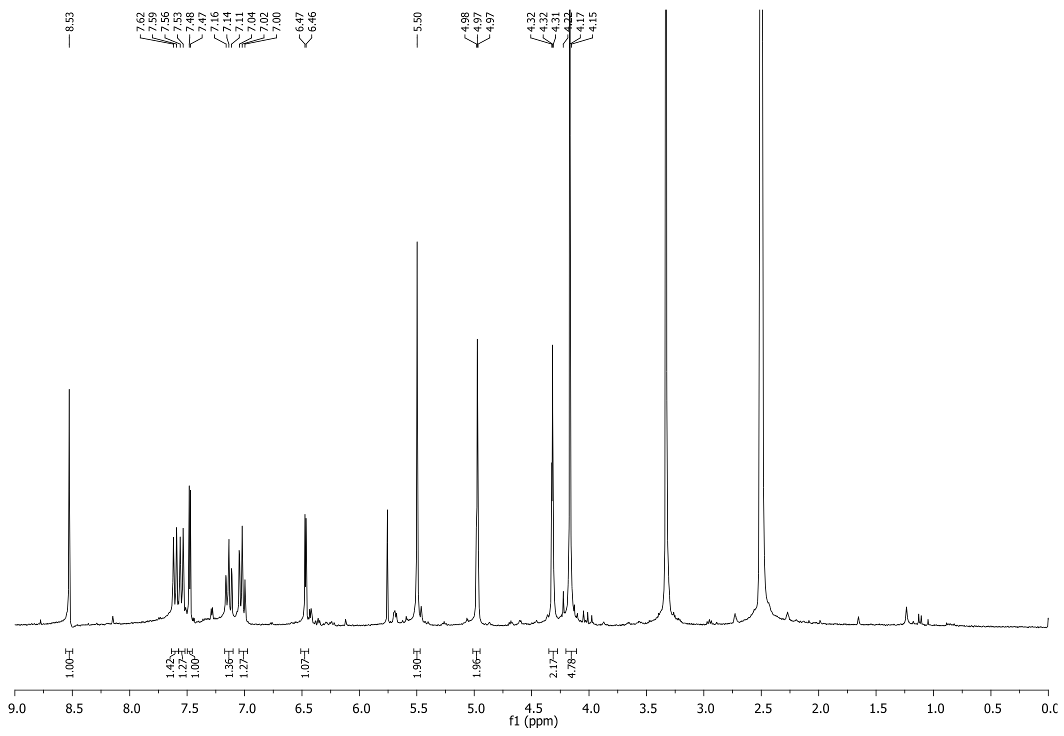

b)

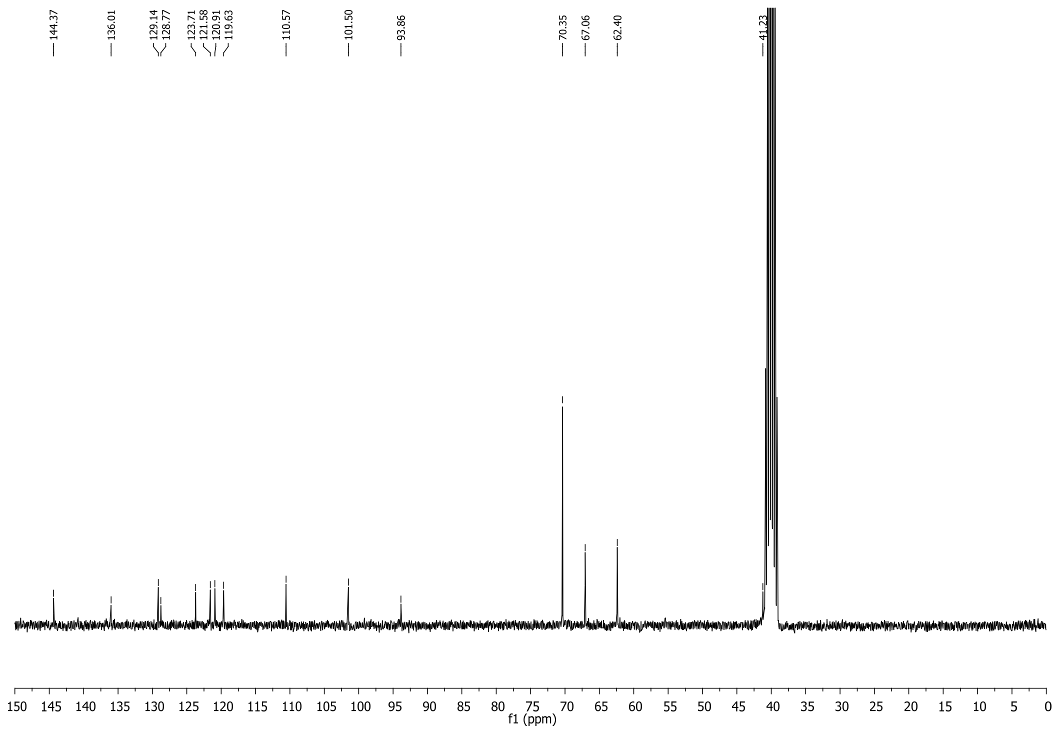

Figure S23. a) ^{1}H i b) ^{13}C-NMR of **25a**

a)

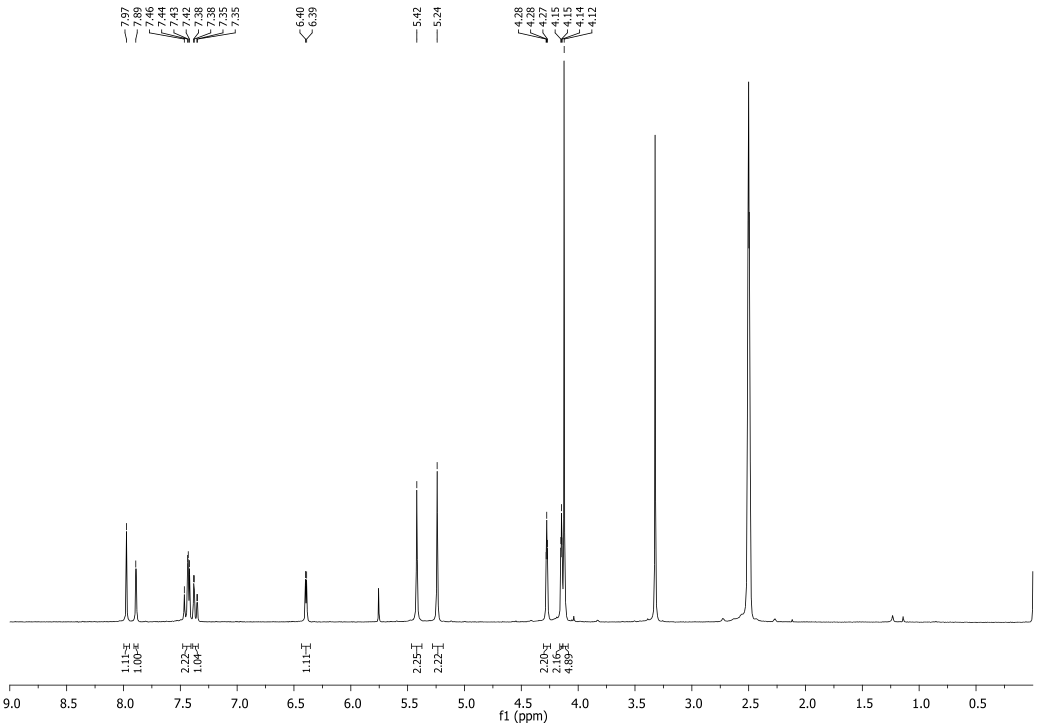

b)

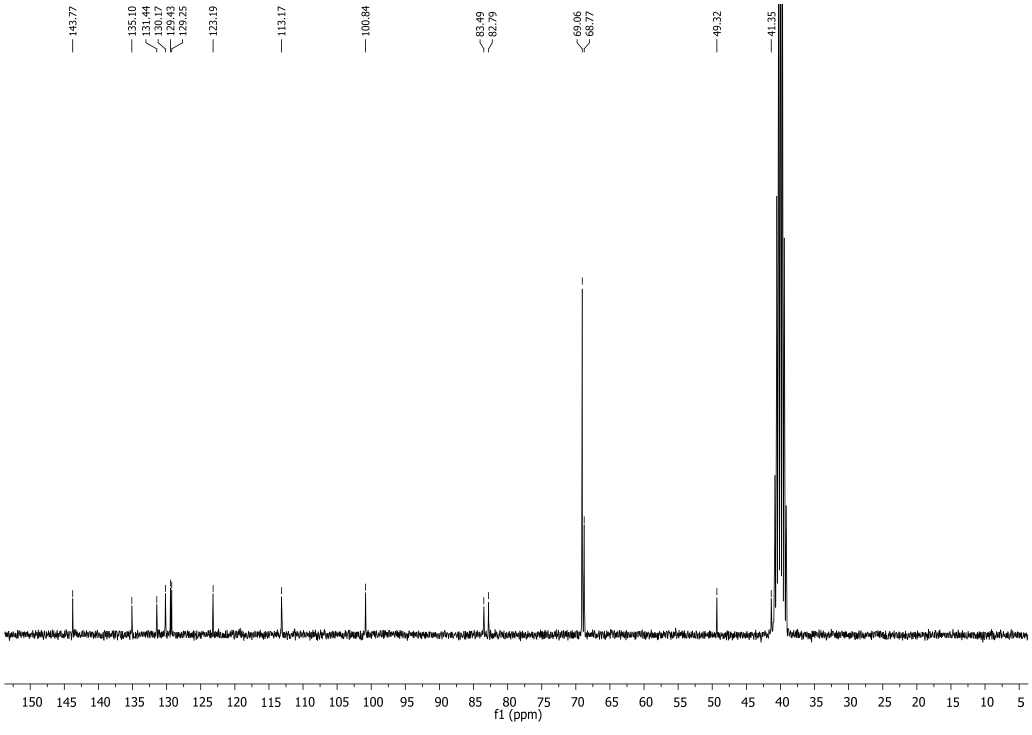

Figure S24. a) ^{1}H i b) ^{13}C-NMR of **25b**

a)

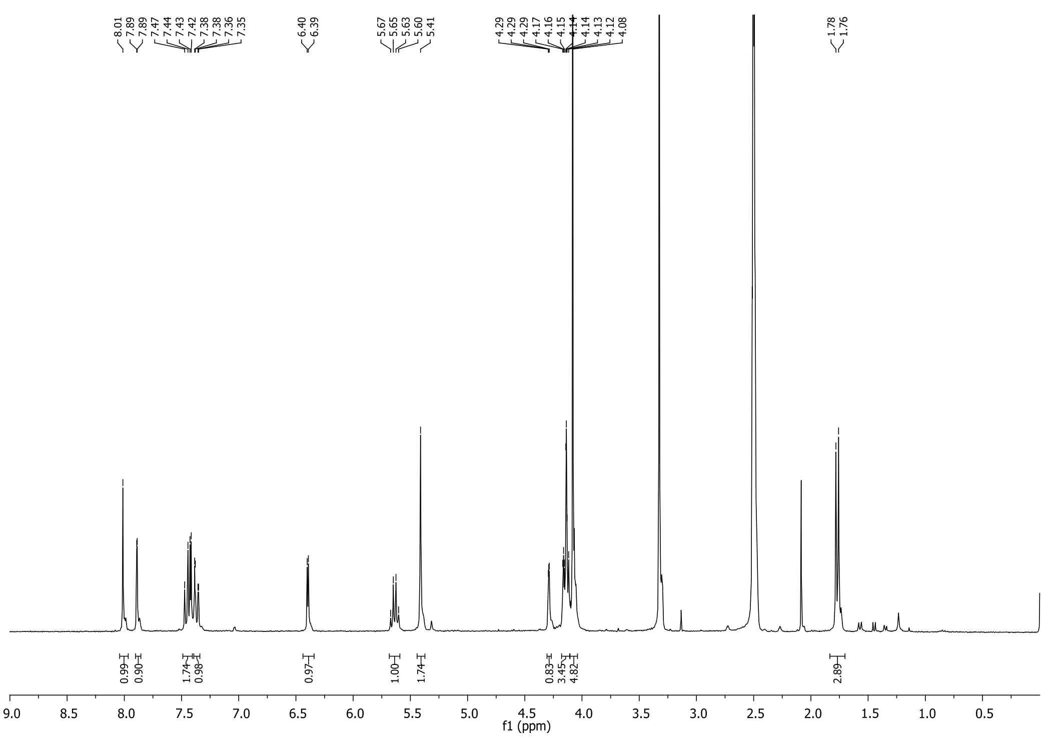

b)

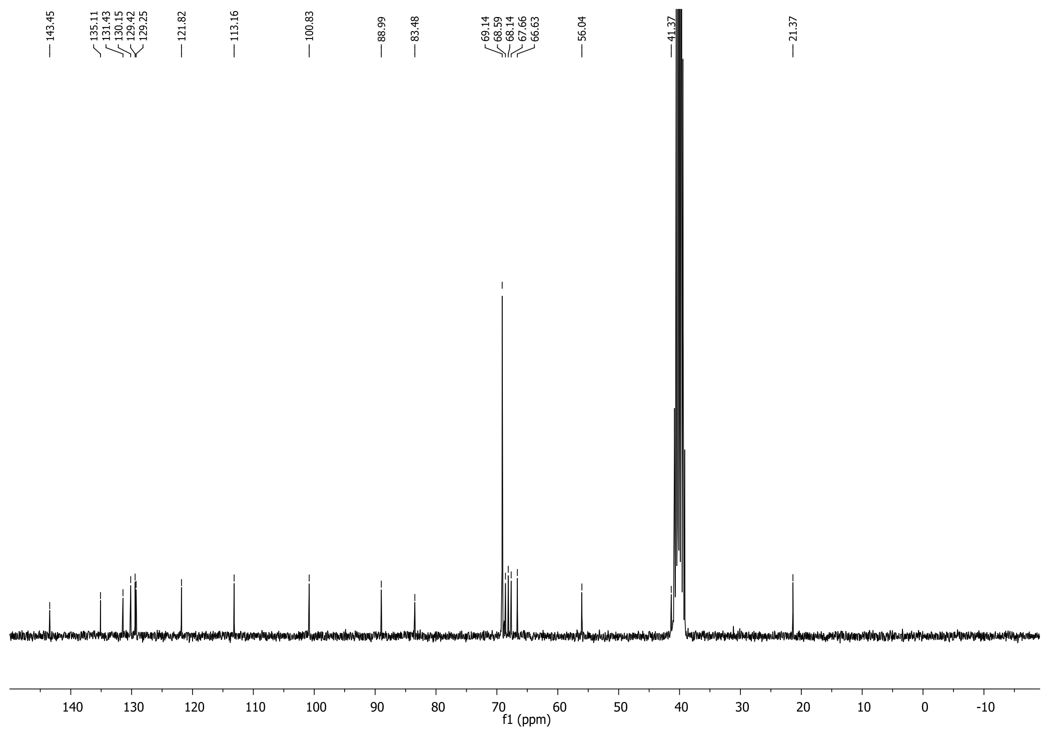

Figure S25. a) ^{1}H i b) ^{13}C-NMR of **25c**

a)

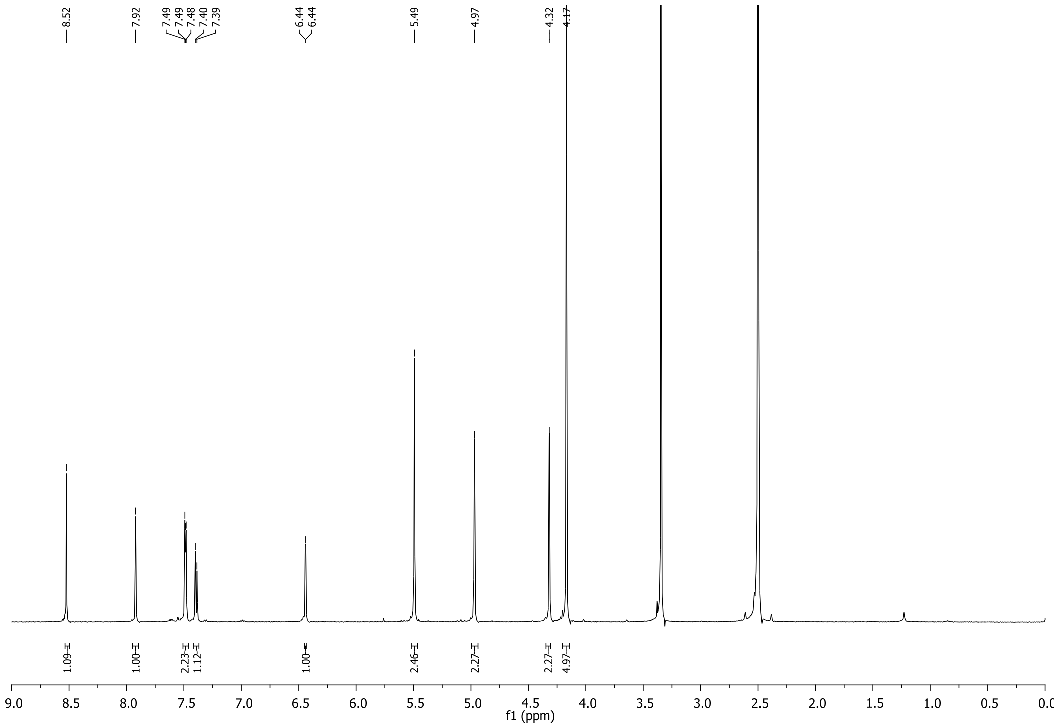

b)

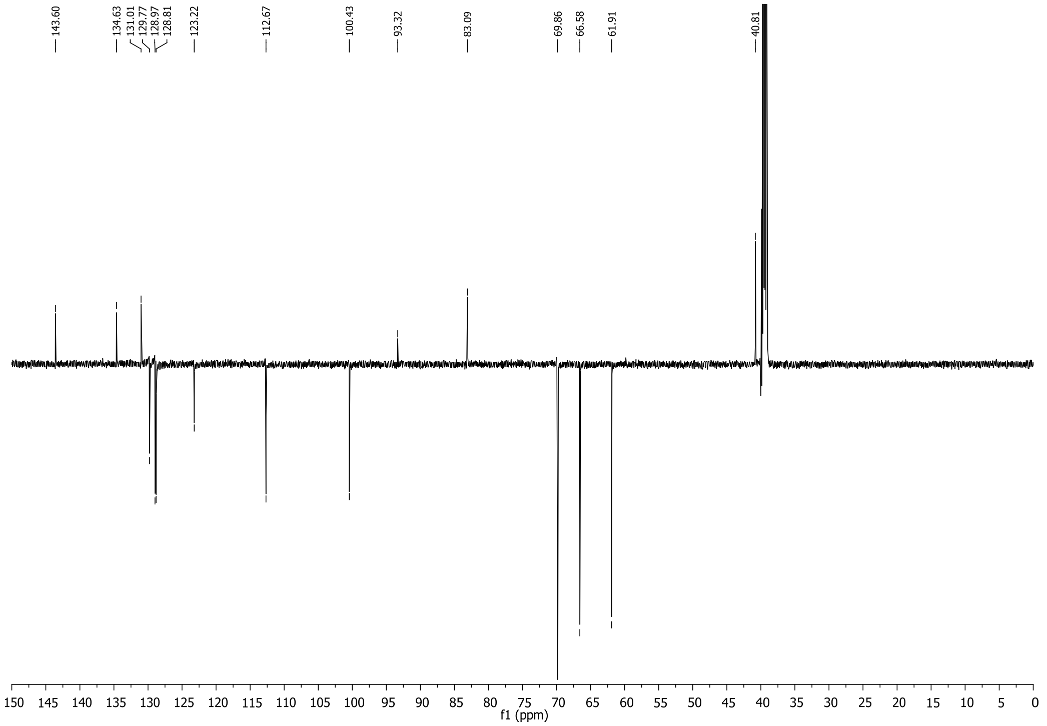

Figure S26. a) ^{1}H i b) ^{13}C-NMR of **26**

a)

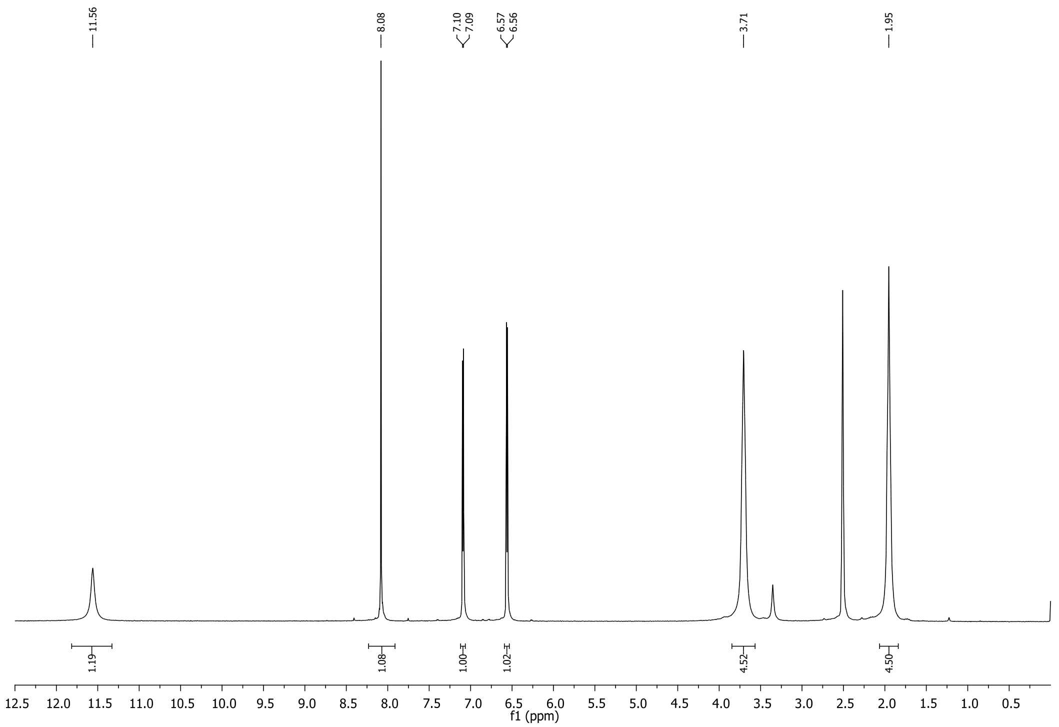

b)

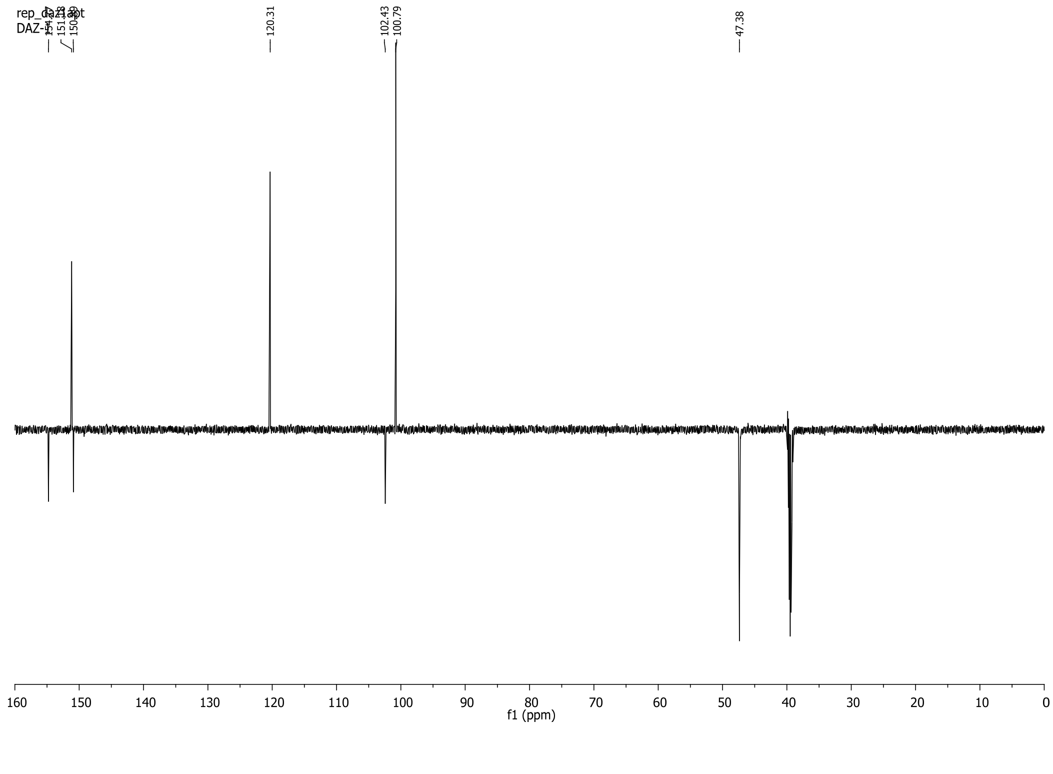

Figure S27. a) ^{1}H i b) ^{13}C-NMR of **27**

a)

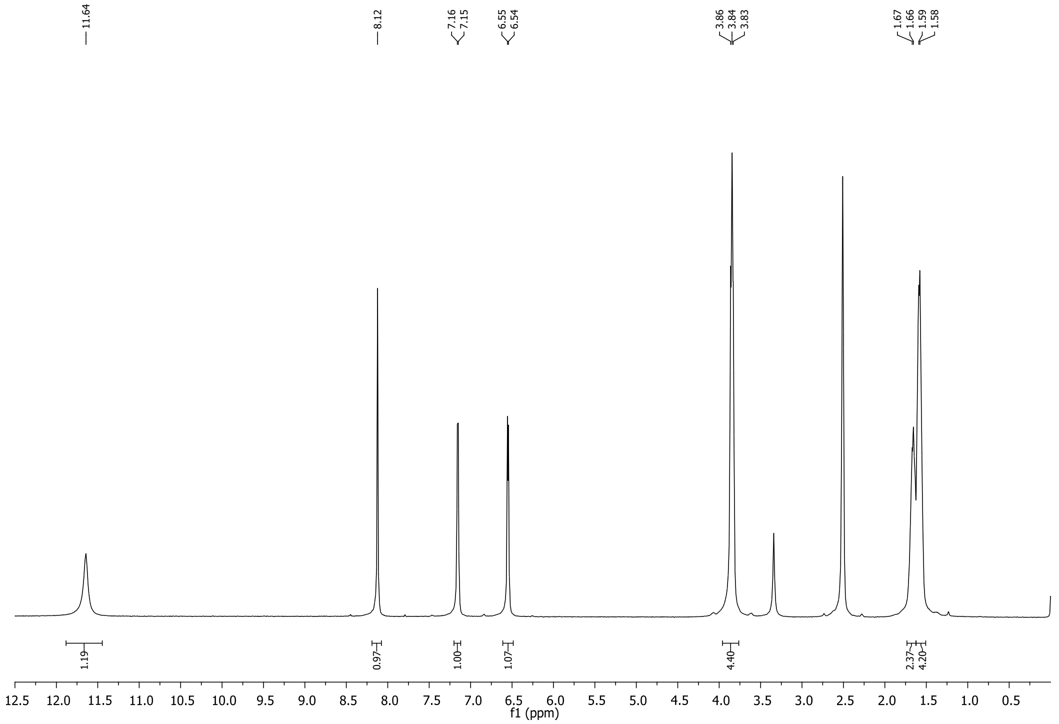

b)

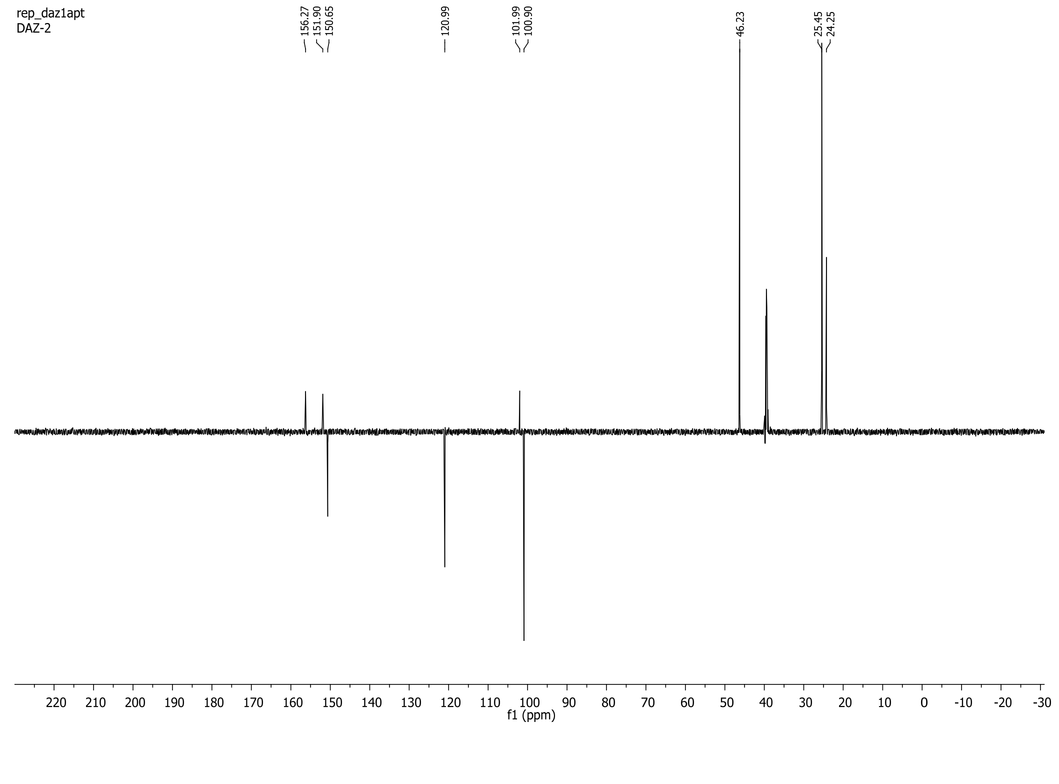

Figure S28. a) ^{1}H i b) ^{13}C-NMR of **30b**

a)

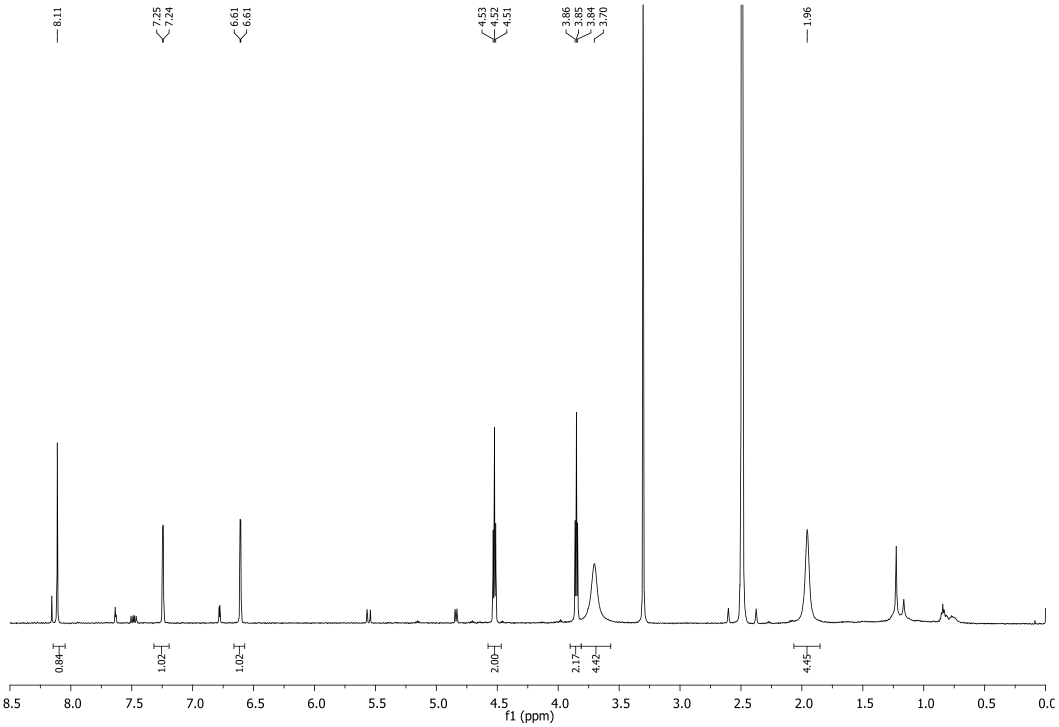

b)

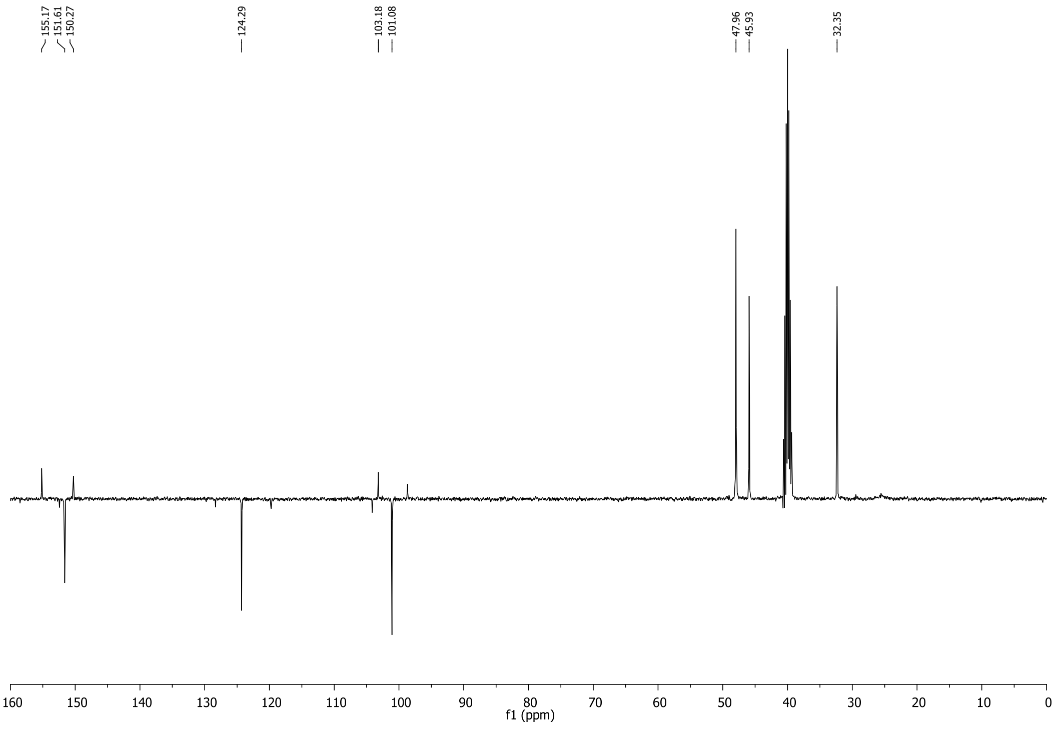

Figure S29. a) ^{1}H i b) ^{13}C-NMR of **30c**

a)

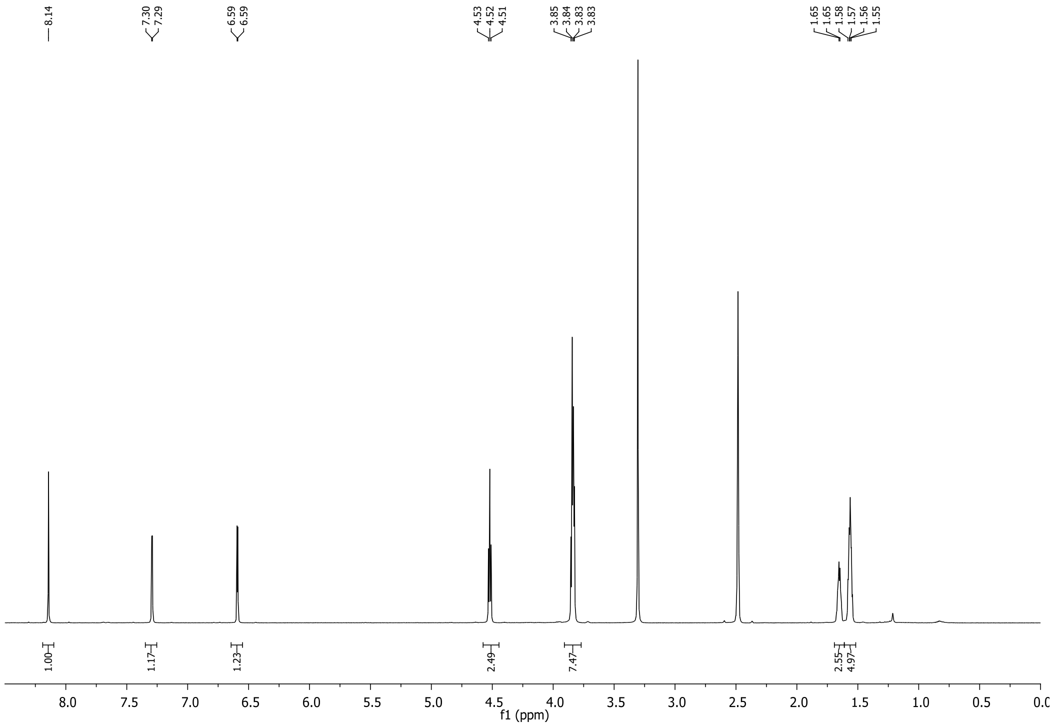

b)

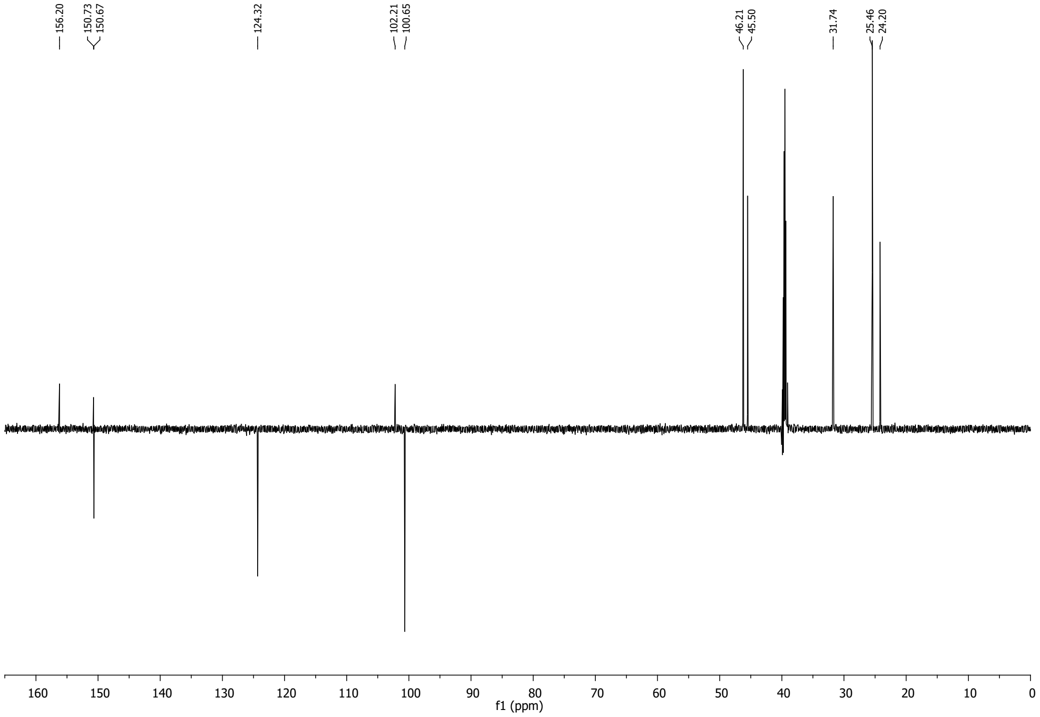

Figure S30. a) ^{1}H i b) ^{13}C-NMR of **31a**

a)

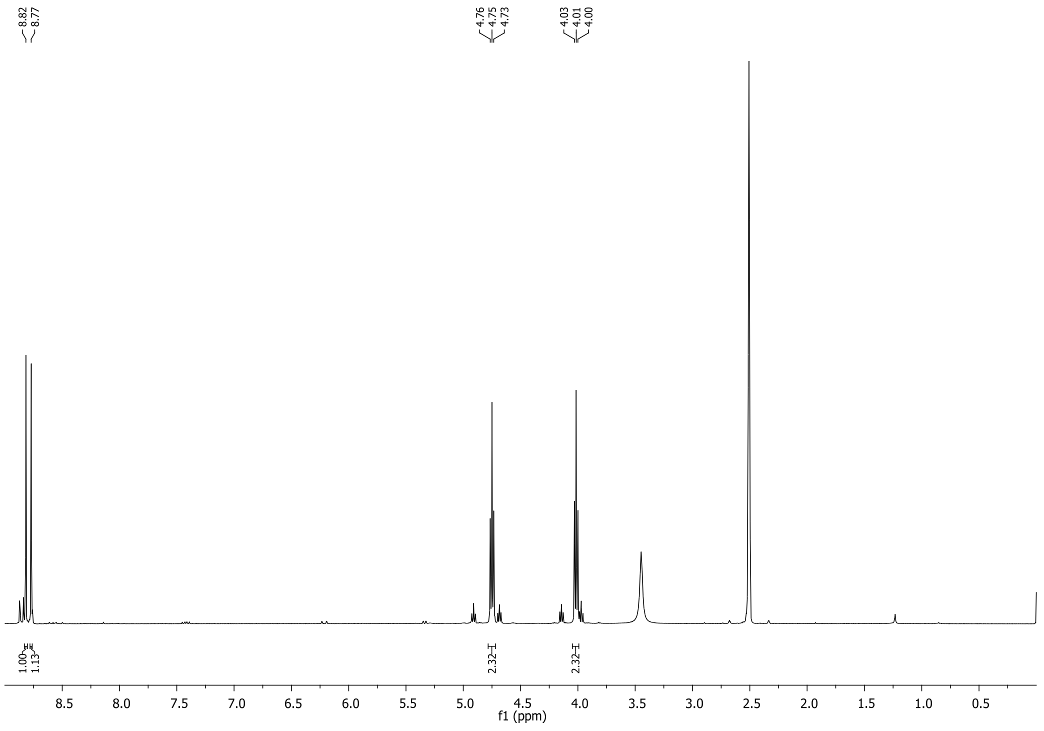

b)

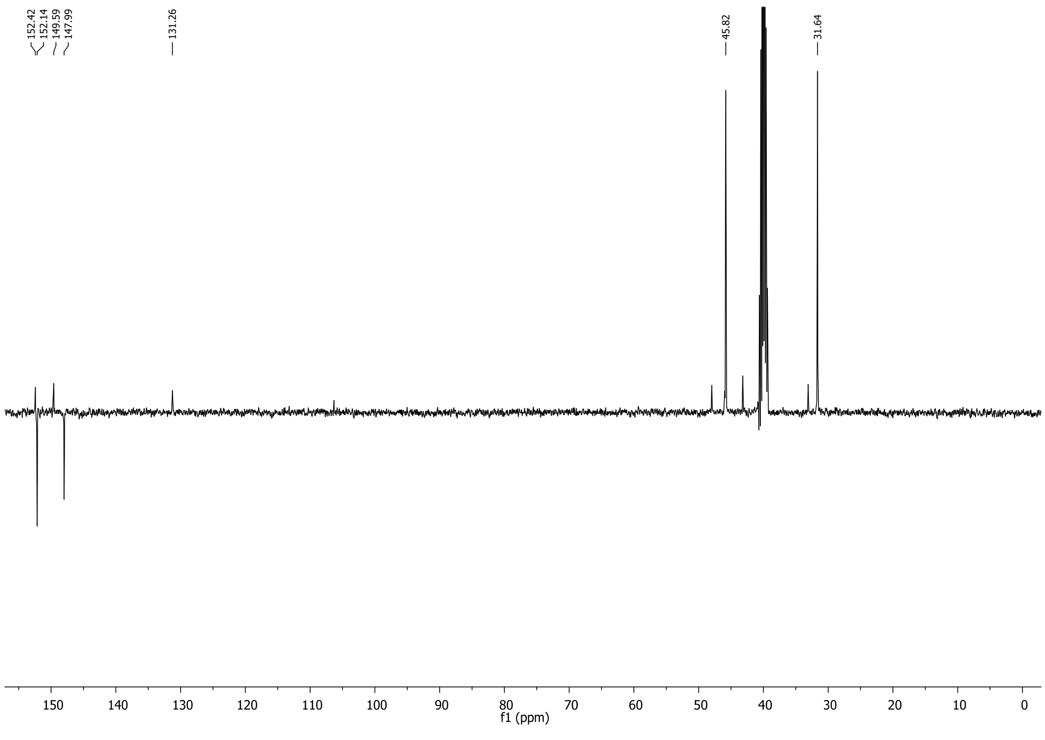

Figure S31. a) ^{1}H i b) ^{13}C-NMR of **31b**

a)

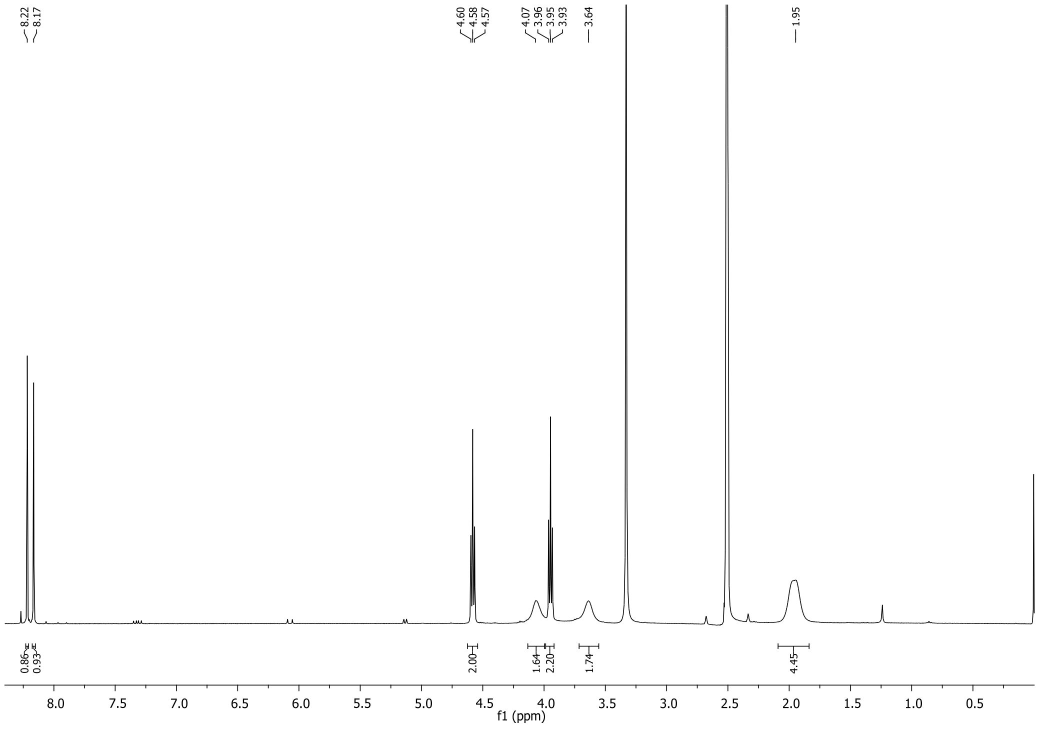

b)

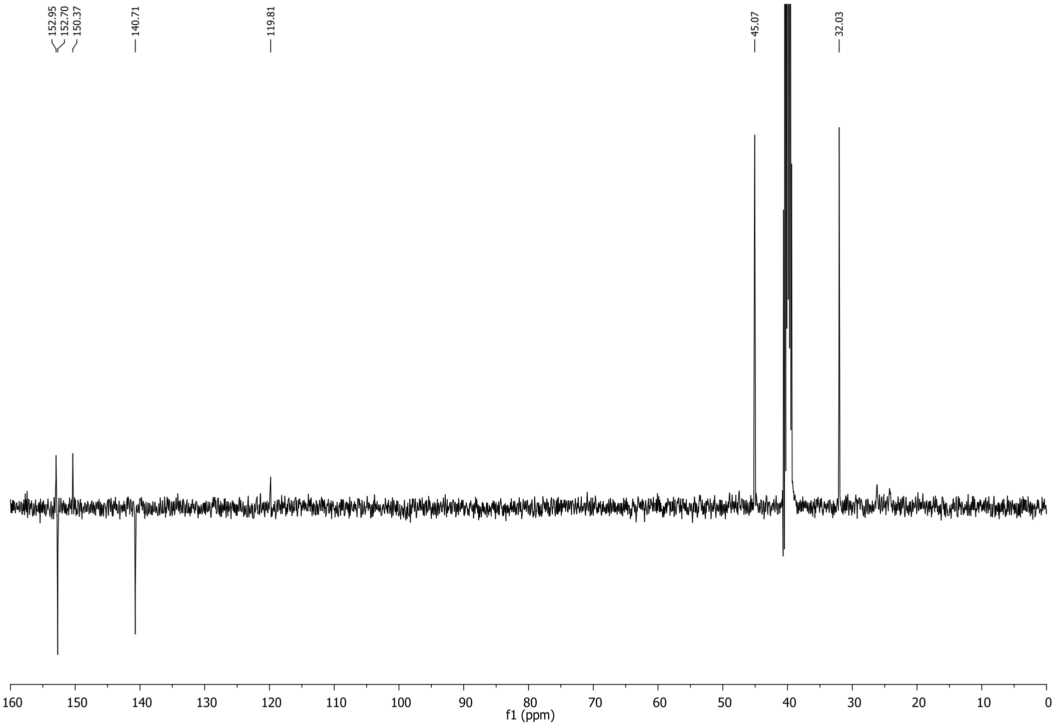

Figure S32. a) ^{1}H i b) ^{13}C-NMR of **31c**

a)

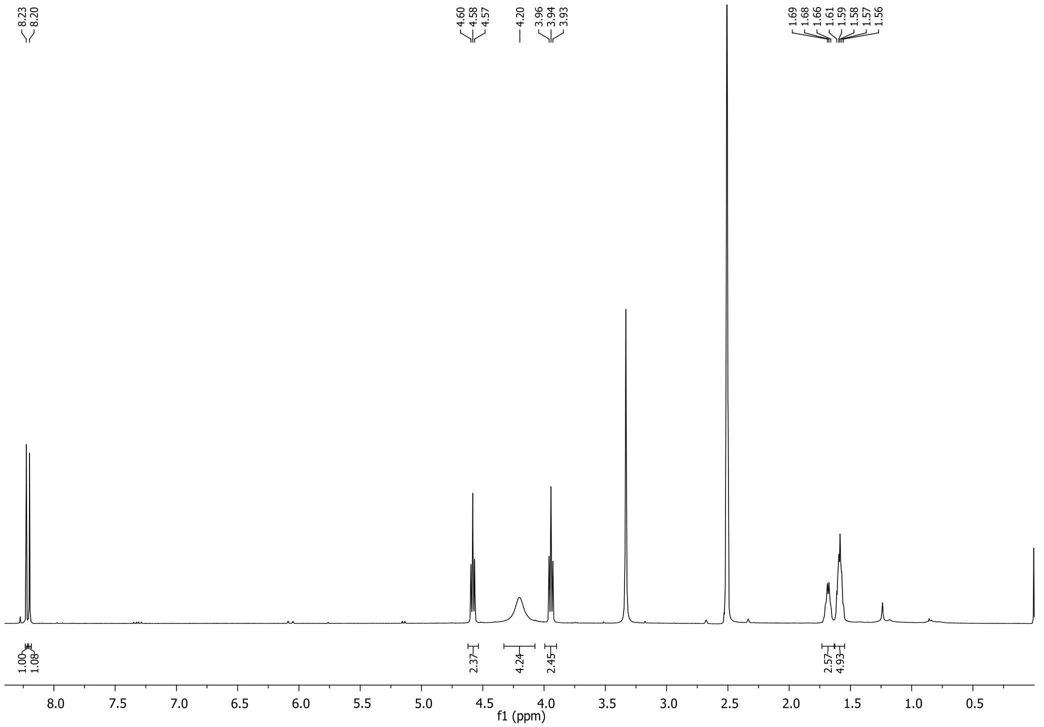

b)

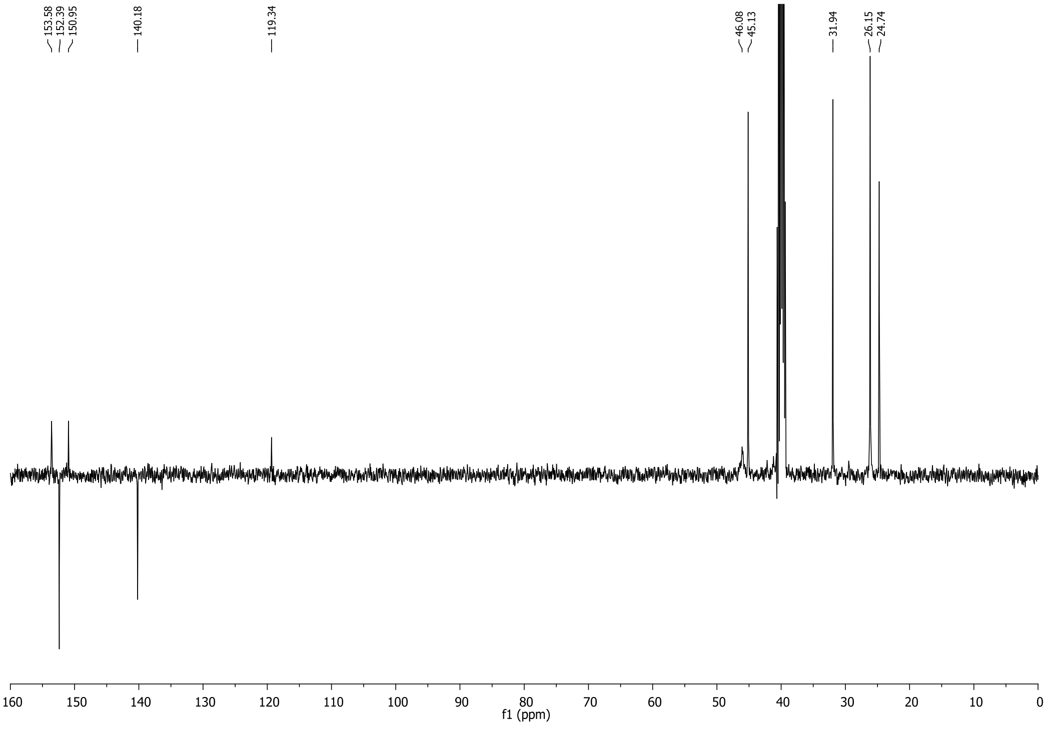

Figure S33. a) ^{1}H i b) ^{13}C-NMR of **34a**

a)

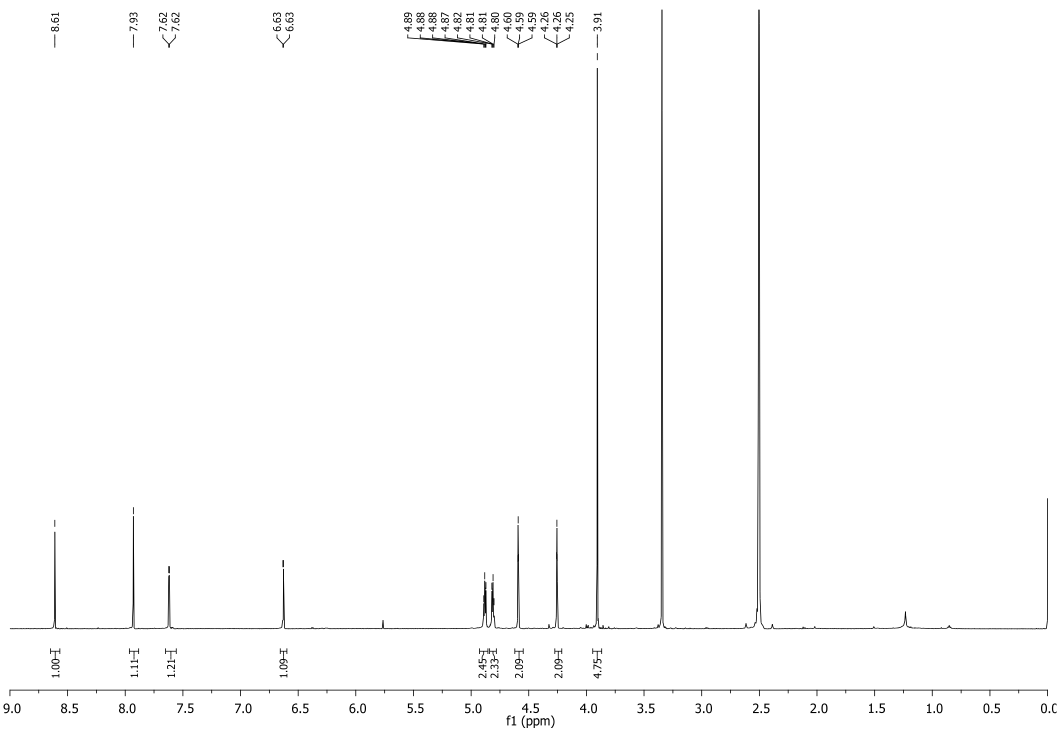

b)

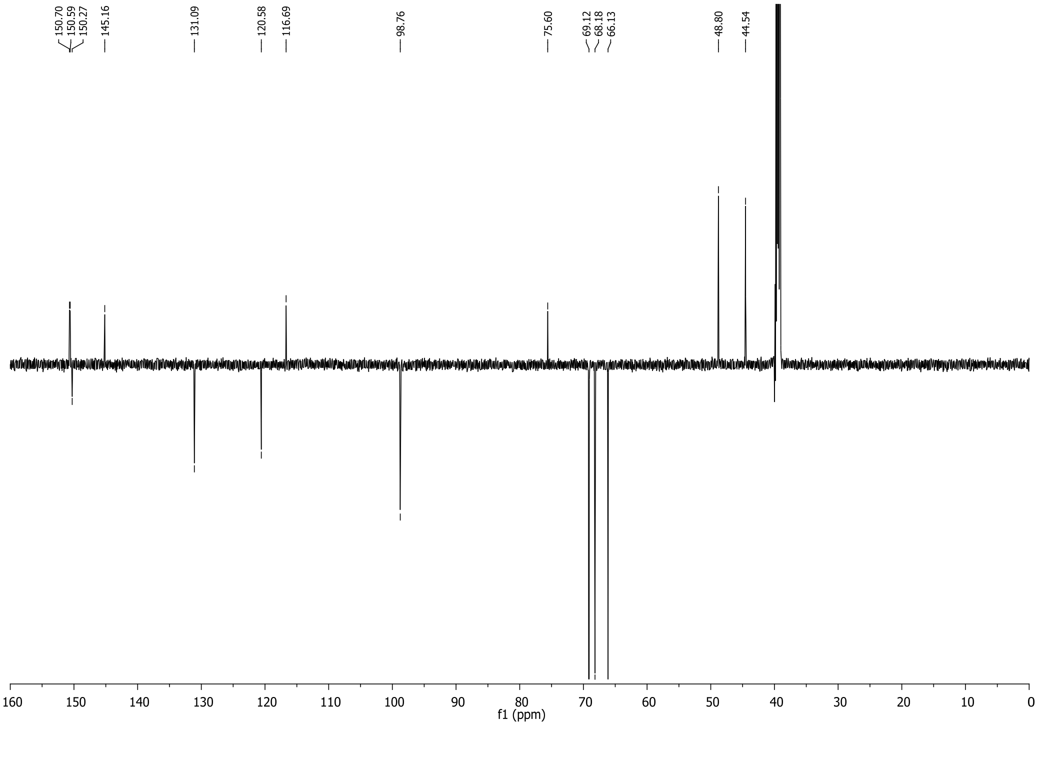

Figure S34. a) ^{1}H i b) ^{13}C-NMR of **34b**

a)

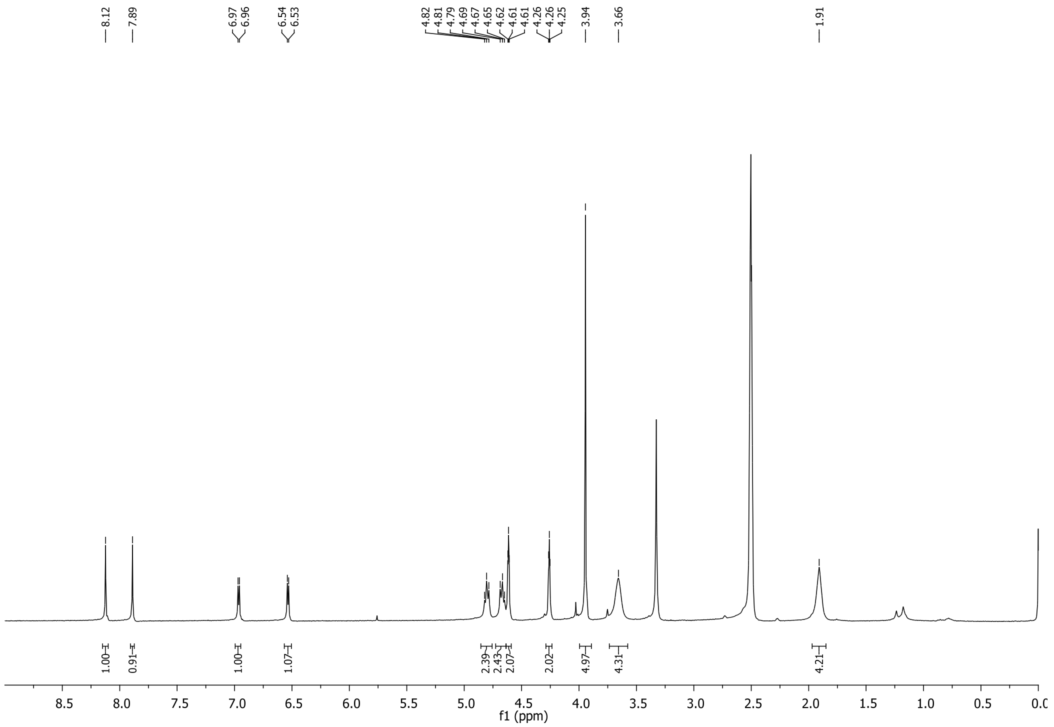

b)

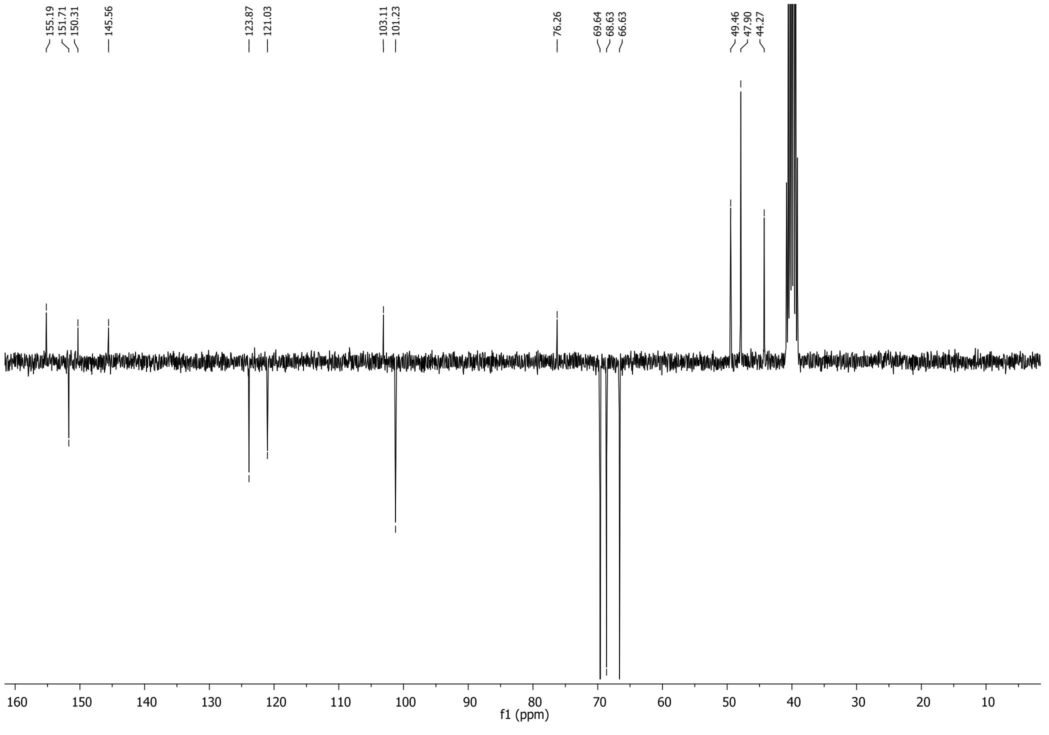

Figure S35. a) ^{1}H i b) ^{13}C-NMR of **34c**

a)

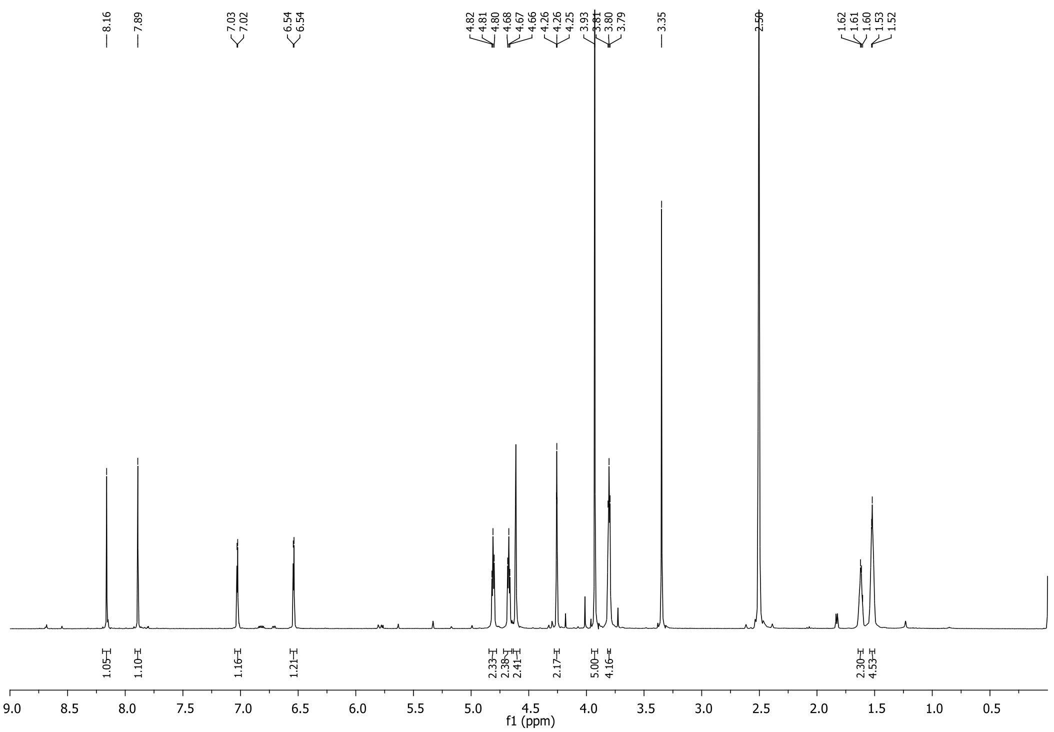

b)

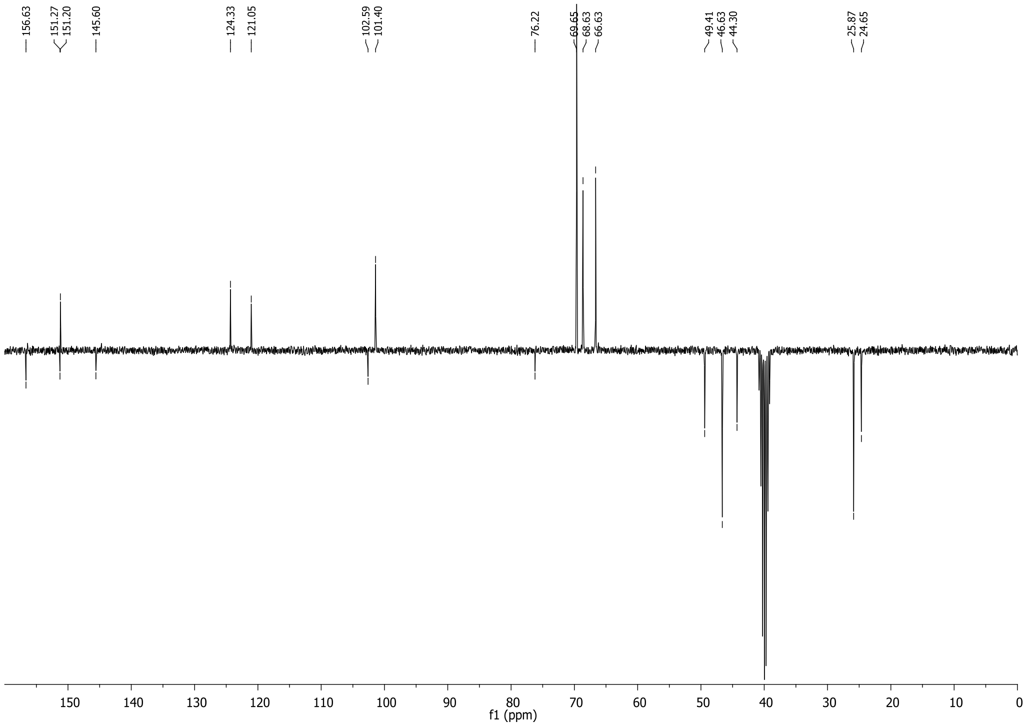

Figure S36. a) ^{1}H i b) ^{13}C-NMR of **35a**

a)

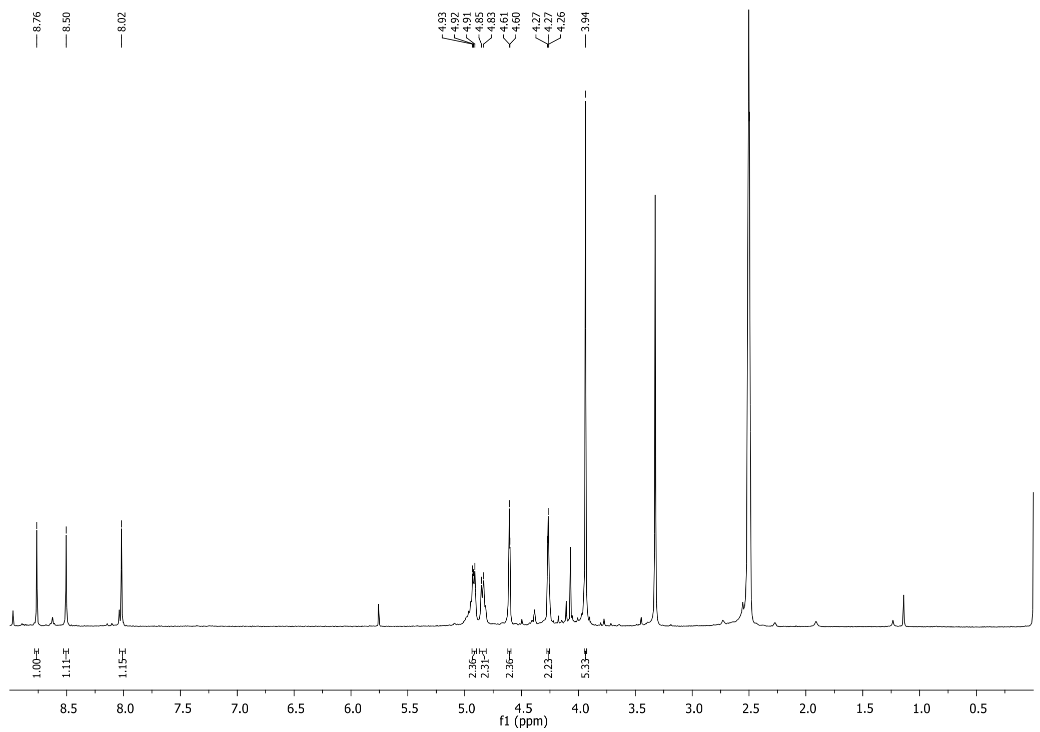

b)

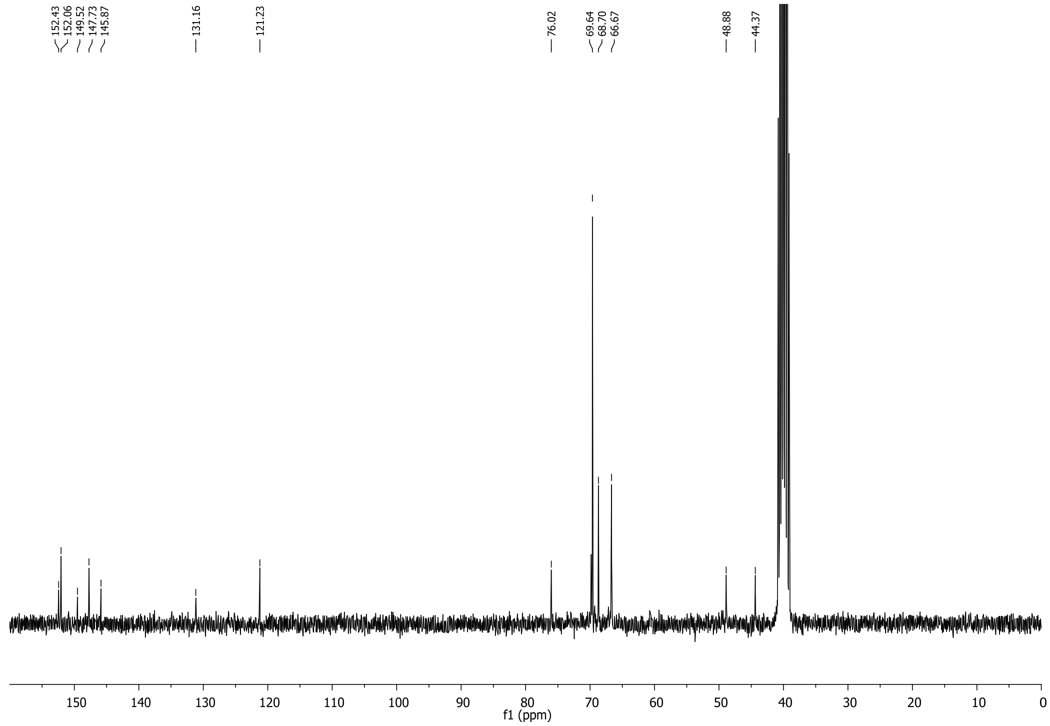

Figure S37. a) ^{1}H i b) ^{13}C-NMR of **35b**

a)

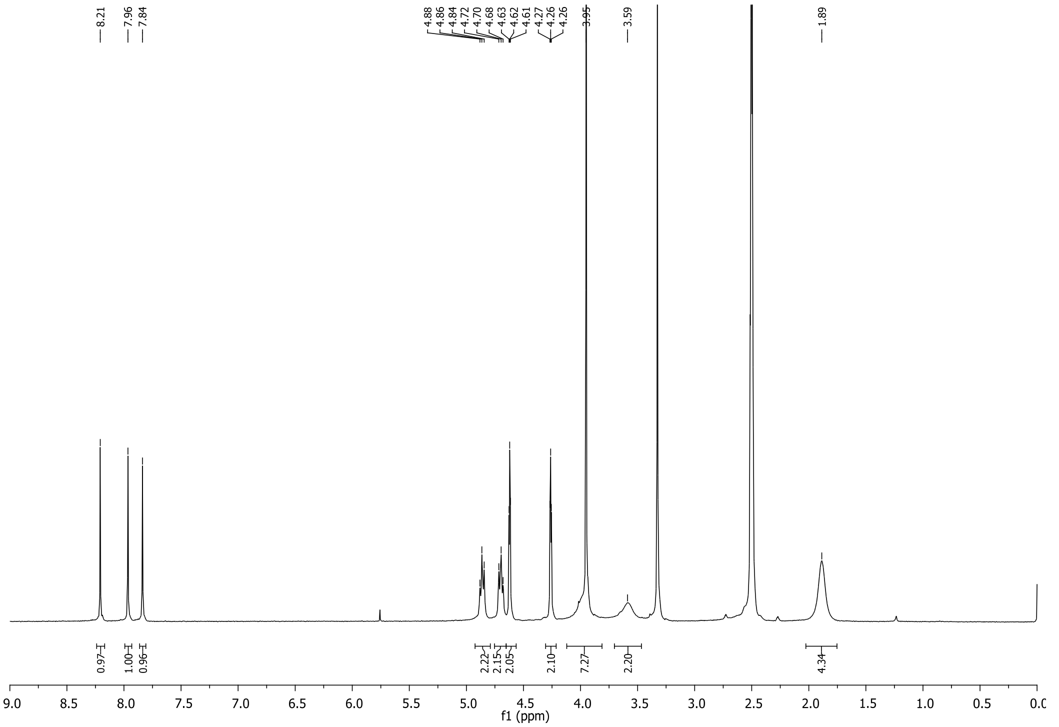

b)

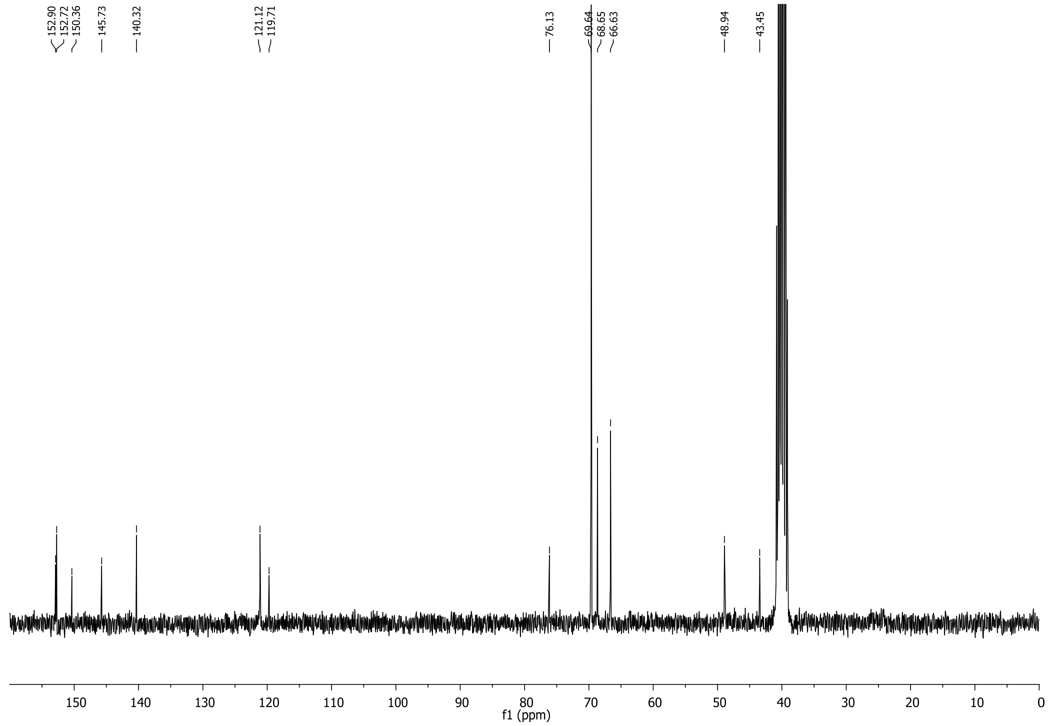

Figure S38. a) ^{1}H i b) ^{13}C-NMR of **35c**

a)

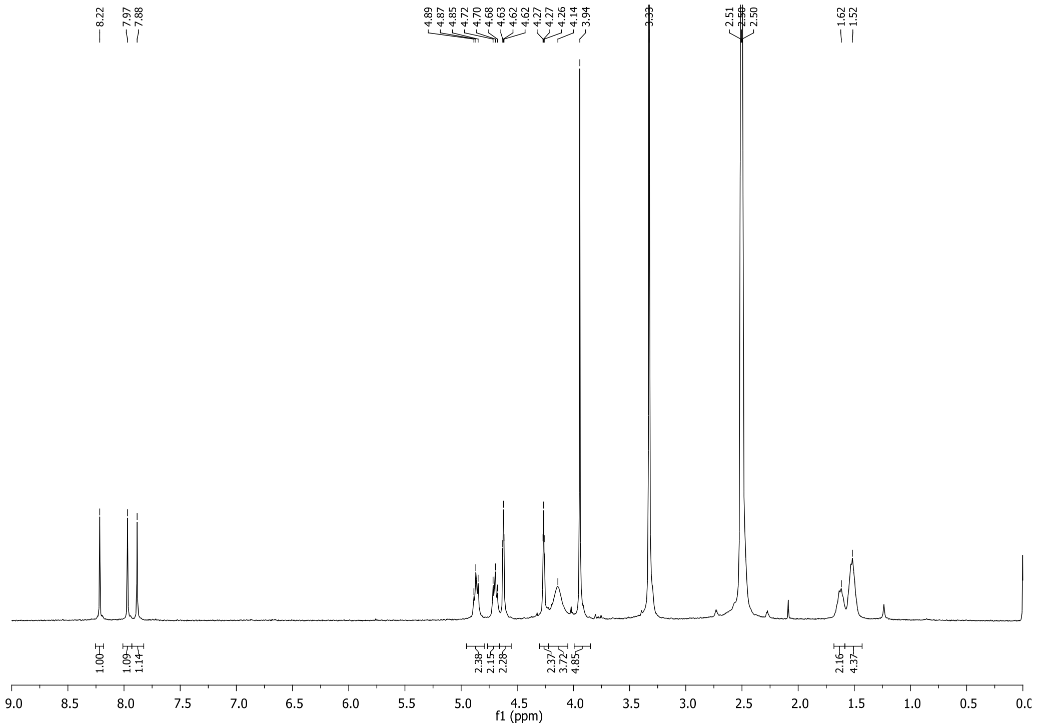

b)

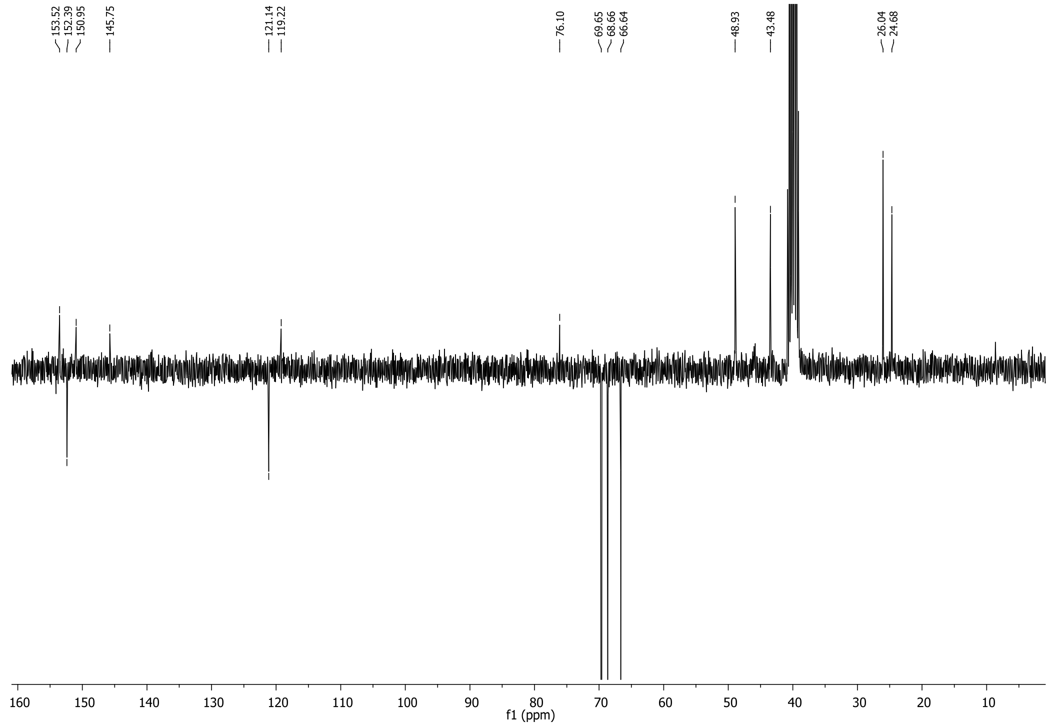

Supplement: Supplementary file 1 [file molecules-25-01570-s001.pdf]
